# Revisiting the immunopathology of congenital disorders of glycosylation: an updated review

**DOI:** 10.3389/fimmu.2024.1350101

**Published:** 2024-03-14

**Authors:** Carlota Pascoal, Rita Francisco, Patrícia Mexia, Beatriz Luís Pereira, Pedro Granjo, Helena Coelho, Mariana Barbosa, Vanessa dos Reis Ferreira, Paula Alexandra Videira

**Affiliations:** ^1^ Associate Laboratory i4HB - Institute for Health and Bioeconomy, NOVA School of Science and Technology, Universidade NOVA de Lisboa, Caparica, Portugal; ^2^ UCIBIO– Applied Molecular Biosciences Unit, Department of Life Sciences, NOVA School of Science and Technology, Universidade NOVA de Lisboa, Caparica, Portugal; ^3^ CDG & Allies-Professionals and Patient Associations International Network, Caparica, Portugal; ^4^ UCIBIO – Applied Molecular Biosciences Unit, Department of Chemistry, NOVA School of Science and Technology, Universidade NOVA de Lisboa, Caparica, Portugal

**Keywords:** congenital disorders of glycosylation, PMM2-CDG, inborn errors of immunity, immune response, host-pathogen interactions, cell adhesion molecules

## Abstract

Glycosylation is a critical post-translational modification that plays a pivotal role in several biological processes, such as the immune response. Alterations in glycosylation can modulate the course of various pathologies, such as the case of congenital disorders of glycosylation (CDG), a group of more than 160 rare and complex genetic diseases. Although the link between glycosylation and immune dysfunction has already been recognized, the immune involvement in most CDG remains largely unexplored and poorly understood. In this study, we provide an update on the immune dysfunction and clinical manifestations of the 12 CDG with major immune involvement, organized into 6 categories of inborn errors of immunity according to the International Union of Immunological Societies (IUIS). The immune involvement in phosphomannomutase 2 (PMM2)-CDG - the most frequent CDG - was comprehensively reviewed, highlighting a higher prevalence of immune issues during infancy and childhood and in R141H-bearing genotypes. Finally, using PMM2-CDG as a model, we point to links between abnormal glycosylation patterns in host cells and possibly favored interactions with microorganisms that may explain the higher susceptibility to infection. Further characterizing immunopathology and unusual host-pathogen adhesion in CDG can not only improve immunological standards of care but also pave the way for innovative preventive measures and targeted glycan-based therapies that may improve quality of life for people living with CDG.

## 1 Glycosylation: a key process in proteome diversity and immune function

For several decades, molecular biology research has been guided by the paradigm that biological information flows from DNA to RNA and to protein. Today it is acknowledged that glycosylation plays a vital role in all species, and that its study is becoming increasingly important to comprehend the entirety of the cellular context (1). Glycosylation is an essential biological process that modulates cell function and molecular stability found in nearly all known living organisms (2, 3). It is a post-translational modification where sugar-chains are assembled, processed, and commonly attached to proteins or lipids, through glycosidic links (1). The sugar components of the glycoproteins have been known to modulate several properties of the parent protein, protecting them from proteolysis, regulating their stability and modifying the protein conformation. In addition, glycans can mediate various physiological functions, as they contribute to structural organization, energy metabolism and are information carriers since their patterns can be recognized by other proteins (4).

Two main types of protein glycosylation exist: N-glycosylation and O-glycosylation (1). N-glycosylation is the covalent attachment of glycans to a protein at specific asparagine (Asn) residues. Its biosynthesis is initiated in the endoplasmic reticulum (ER) membrane, where a precursor oligosaccharide is assembled on a resident lipid carrier (5, 6). An oligosaccharyltransferase complex then transfers the glycan to a target protein via a N-glycosidic linkage of a N-acetylglucosamine (GlcNAc) to an Asn residue in the consensus amino acid sequence Asn-X-serine (Ser)/threonine (Thr), in which “X” is any amino acid except for proline (Pro) (7). The subsequent stage includes the trimming and processing in the ER and Golgi, converting a limited repertoire of N-glycans into a vast array of mature and complex N-glycans (8). O-glycosylation, on the other hand, is the attachment of glycans initiated by N-acetylgalactosamine (GalNAc) to the hydroxyl group of Ser or Thr residues in proteins, usually initiated in the Golgi by N-acetylgalactosaminyltransferases (GalNAcTs) (8). Subsequently, sequential enzymatic elongation steps elongate this Ser/Thr-GalNAc structure to form longer and more complex structures (9). Both N- and O-glycosylation processes can be further modified by sialylation, sulfation, acetylation, fucosylation, and polylactosamine-extension.

Glycosylation introduces considerable variety to the proteome with effects on protein functionality, localization, solubility, and stability, thereby contributing substantially to biological complexity (10). It is important for cell-cell and cell-matrix interactions, with glycans lining the cell surface as mediators of intra and extracellular communication, signaling, immune recognition, and pathogen detection (11). The vast diversity of glycans, resulting from saccharide position and stereochemistry options, contribute not only to the complexity but also to the specificity of the mechanisms and interactions outlined above (10).

In the immune function, glycosylation has a crucial role as most of the molecules involved in the immune response are glycoproteins (12). Immunoglobulins (Ig), adhesion molecules, cytokines, chemokines, complement proteins, and pathogen recognition receptors, such as Toll like receptors, are heavily glycosylated (13–16). Specifically, N-glycans decorate the α and β chains of the T cell receptor and the heavy chain of the MHC class I complex (17–19). Most proteins present multiple glycosylation sites and can be decorated with both N- and O- glycans, such as all Ig classes, and immunoregulators such as CD45 (20–22).

Glycosylation plays a dual role in pathogen infection. On one hand, glycans can prevent microbial attachment and invasion, by reinforcing the physical barriers that are part of the innate immune response. This is exemplified by mucins, high molecular-weight glycoproteins with extensive O-glycosylation, that form a viscous protective barrier between the epithelial cells and microorganisms (23). Conversely, glycosylation facilitates infection and helps pathogens escape the immune defenses, as pathogens interact with host glycans through glycan-binding proteins (e.g., pili), promoting adherence or internalization (24, 25). Furthermore, microbial patterns are also glycans or glycoconjugates that often mimic the host carbohydrates, favoring the escape from the immune surveillance (26).

Deregulation of glycosylation pathways can then be at the root of critical changes in physiological processes, such as the immune function, and is commonly found to be associated and to modulate the progression of various disorders. For instance, IgG with N-glycosylation lacking terminal galactose and sialic-acid linkages has been associated with rheumatoid arthritis (27). Such glycosylation modifications can trigger an inflammatory response through recognition by complement mannose-binding lectins. Inflammatory bowel disease (IBD), whose full etiology remains unclear, has also been associated with aberrant glycosylation. Disruption of the binding and signaling of the glycan-binding protein galectin-1 has been linked to increased susceptibility for colitis and disruption of intestinal homeostasis (28). Therefore, the balance between health and disease requires both glycosylation and modulation of glycan-binding proteins.

## 2 Exploring glycosylation defects in congenital diseases

Congenital disorders of glycosylation (CDG) represent a group of 163 rare inherited metabolic defects encompassing 193 different phenotypes stemming from abnormalities in glycosylation biosynthesis and processing (29). These disorders can be classified into four main categories based on their underlying defect: i) N-linked glycosylation (e.g., MAN2B2-, PGM3-, ALG12-CDG), ii) O-linked glycosylation (e.g., B3GALT6-, XYLT2-, and EXTL3-CDG), iii) combined N- and O- linked/multiple glycosylation pathways (most prevalent with number of phenotypes), (e.g., AT6AP1-, ATP6V1A-, B4GALT1-CDG) and iv) lipid and glycosylphosphatidylinositol (GPI) anchor defects (e.g., PIGA-, PIGB-, PIGW-CDG) (29–31). As a result of the vast molecular diversity, this group of diseases exhibits a remarkable genetic and clinical heterogeneity within each and between CDG. This variability is illustrated by numerous system and organs, such as the varying degrees of severity of intellectual disability and developmental delay, observed, for example, in PMM2-, PIGA- and ALG12-CDG (32). Furthermore, even within patients with the same genetic variant, there are differences in clinical symptomology and progression, as demonstrated in GNE-CDG (33). This issue reflects the lack of biochemical biomarkers, where even the commonly used transferrin isoelectric focusing (TIEF) assay can only screen for a limited number of CDG (34). Furthermore, even within the identifiable CDG, some patients might not show different profiles compared to healthy individuals (35, 36). Nevertheless, some potential markers have been identified for few CDG and alternative approaches like glycomics have been explored (34). As we delve deeper into CDG mechanisms, there are clinical manifestations such as those derived from immune system, which accentuate the complexity and challenges our current understanding of the role of glycans. While extensive research has been dedicated to unravelling how glycans govern immune cell-cell and receptor-ligand interactions, only few studies endeavored to elucidate these mechanisms in the context of CDG and established a link with diverse clinical observations.

In the following sections, we delve into specific CDG with reported immunological implications. Understanding the immunological aspects of CDG becomes paramount given the multifaceted role of glycosylation in regulating immune responses. The insights gleaned from a revision of literature on the current evidence of immune implications in CDG may have implications beyond CDG, contributing to a broader understanding of the immune response mechanisms and with potential clinical applications.

## 3 Immunological burden in congenital disorders of glycosylation

The immunological burden of CDG is well-mirrored by their clinical heterogeneity. In view of the intricate interplay between glycosylation and immune responses (see section “Glycosylation: a key process in proteome diversity and immune function”), it is reasonable to expect that among the 163 known CDG, some would exhibit immune defects. Previous studies referred that 23 CDG had minor (less than 50% of patients) or major (more than 50% of patients) immunological involvement according to the prevalence of immunological clinical manifestations (e.g., infectious, inflammatory, allergic, or autoimmune events, biochemical or functional alterations and abnormal responses to vaccination) (37, 38). However, the predominance and relevance of these immune events compared to the average healthy population remain unclear, underscoring the need to revisit the list of CDG with immunological burden.

**Figure 1:**
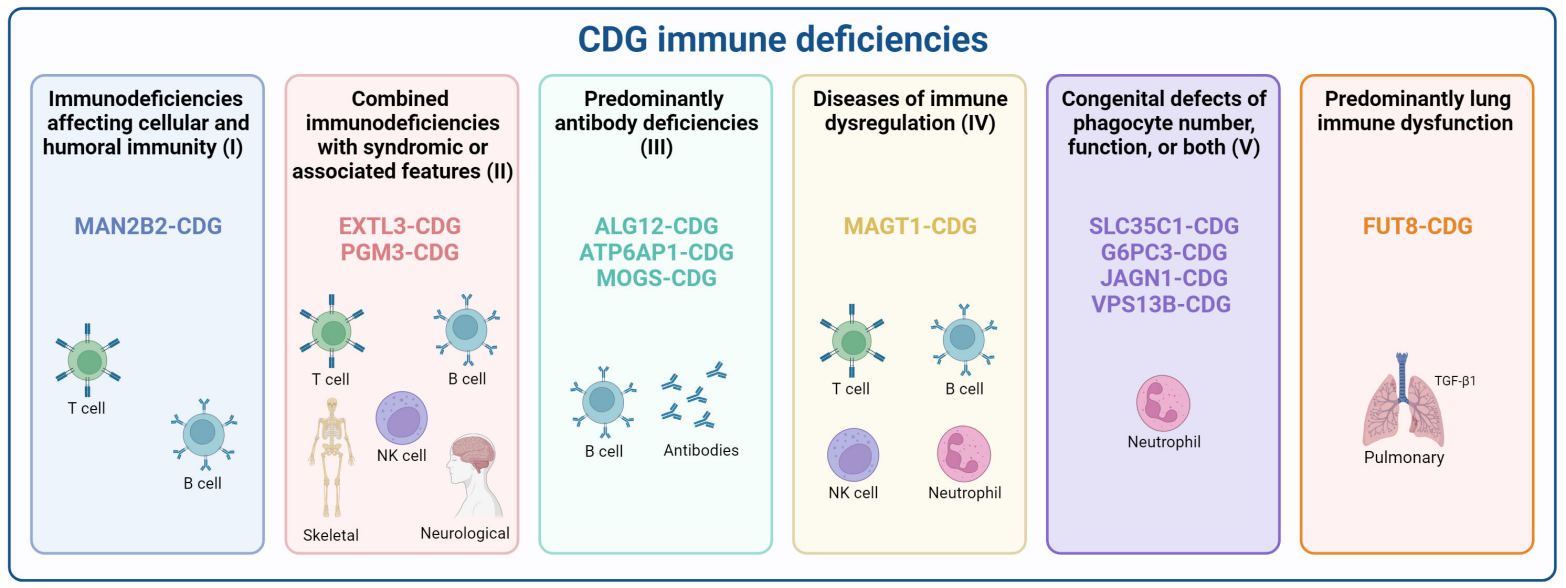
Classification of CDG according to inborn errors of immunity. CDG presenting immune deficiencies were categorized according to the recent International Union of Immunological Societies (IUIS) classification as (I) Immunodeficiencies affecting cellular and humoral immunity (MAN2B2-CDG), (II) Combined immunodeficiencies with syndromic or associated features (EXTL3-, and PGM3-CDG), (III) predominantly antibody deficiencies (ALG12-, ATP6AP1-, and MOGS-CDG), (IV) Diseases of immune dysregulation (MAGT1-CDG), (V) Congenital defects of phagocytes (SLC35C1-, G6PC3-, JAGN1-, and VPS13B-CDG), and a provisional classification for diseases with Predominant lung immune dysfunction (FUT8-CDG). Created using BioRender.com.

Importantly, according to the recently updated classification of the International Union of Immunological Societies (IUIS) (39, 40), 11 CDG have been reported as having immunological dysfunction or manifestations as one of the primary disease hallmarks. These CDG span across the five categories within the Inborn Errors of Immunity classification, based on clinical features, biochemical presentations, and impaired mechanisms. Additionally, we describe FUT8-CDG as a CDG with predominant lung immune dysfunction (**Figure 1**). The details of the inclusion of each of these CDG in these different groups will be detailed below, and their reported immunological manifestations are portrayed in **Supplementary Table 1**.

### 3.1 Immunodeficiencies affecting cellular and humoral immunity

One notable inclusion in this category is MAN2B2-CDG, recently recognized as an inborn error of immunity with the first reported patient presenting immunodeficiency, dysmorphic facial features, coagulopathy, and severe developmental delay (39, 41).

**Figure 2:**
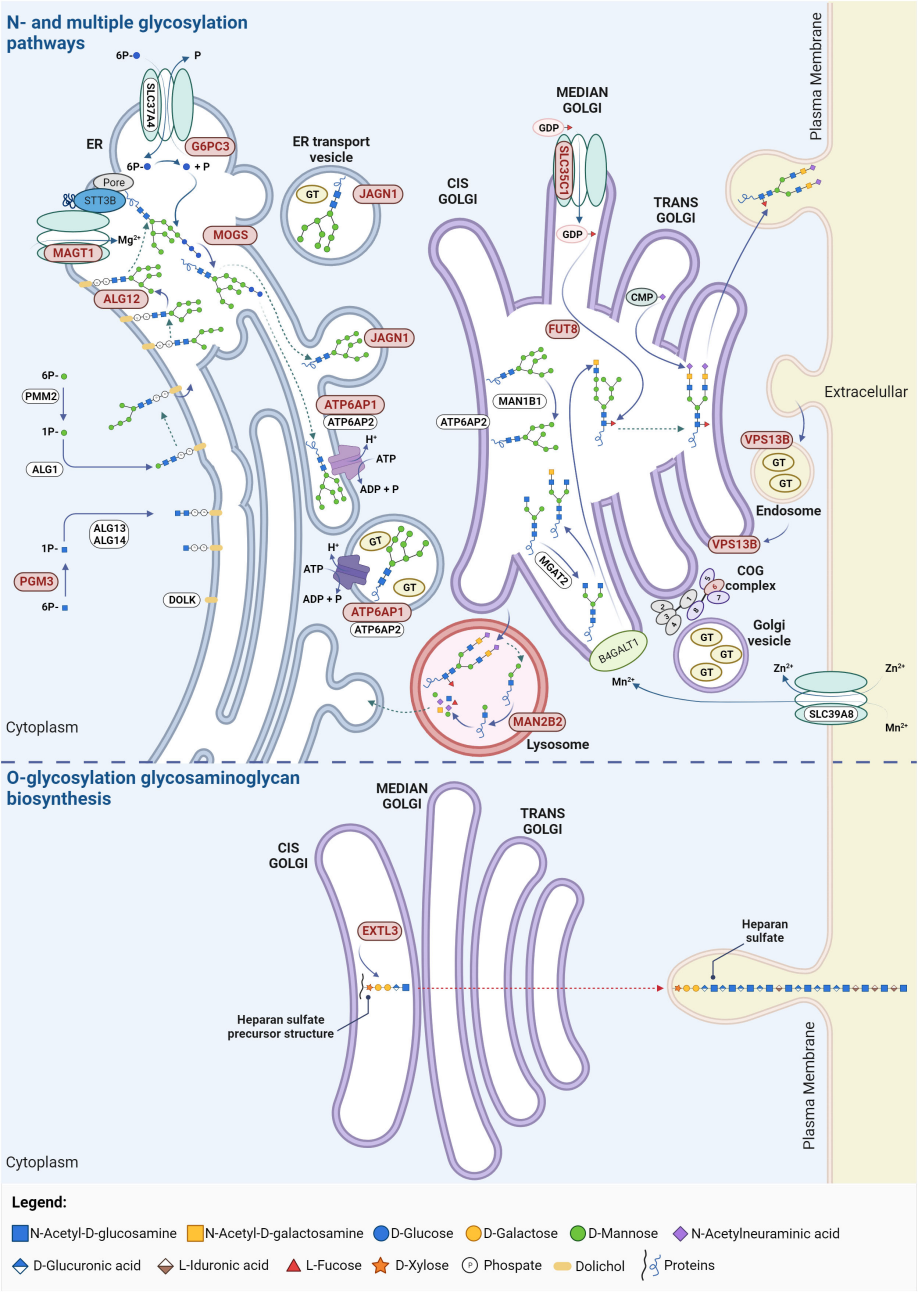
Glycosylation pathways in CDG with immunological involvement. The affected proteins in these CDG participate in N-glycosylation, O-glycosylation (particularly in the synthesis of heparin and heparan sulphate) and multiple glycosylation pathways. Proteins linked to CDG major immunological involvement are highlighted in red. ADP, adenosine diphosphate; ALG, asparagine-linked glycosylation; ATP, adenosine triphosphate; ATP6AP1/2, ATPase H+ transporting accessory protein 1/2; B4GALT1, β-N-acetylglucosaminyl-glycolipid β-1,4-galactosyltransferase; CMP, cystidine-5’-monophosphate; COG, conserved oligomeric Golgi; DOLK, dolichol kinase; ER, endoplasmic reticulum; EXTL3, exostosin like glycosyltransferase 3; FUT8, fucosyltransferase 8; G6PC3, glucose-6-phosphate catalytic subunit 3; GDP, guanosine diphosphate; GT, glycosyltransferase; JAGN1, jagunal homolog 1; MAGT1, magnesium transporter 1; MAN1B1, mannosidase α class 1B member 1; MAN2B2, mannosidase α class 2B member 2; MGAT2, α-1,6-mannosyl-glycoprotein 2-β-N-acetylglucosaminyltransferase; MOGS, mannosyl-oligosaccharide glucosidase; PGM3, phosphoglucomutase 3; PMM2, phophomannomutase 2; SLC, solute carrier family; VPS13B, vacuolar protein sorting 13 homolog. Created using BioRender.com.

MAN2B2 is a mannosidase involved in the second-to-last step of lysosomal degradation of glycoproteins, the cleavage of the α1,6-mannose residue of Man2GlcNAc1 to generate Man1GlcNAc1 (**Figure 2**). MAN2B2 deficiency dysregulate the deglycosylation and monosaccharide recycling process, leading to the accumulation of terminal 1,6-mannose-bearing Man2GlcNAc1 and defective protein N-glycosylation (41). The clinical manifestations in the first reported patient were recurrent vasculitis, arthritis, and infections. The patient showed decreased counts of naïve cytotoxic and helper T cells, but increased effector memory T cells, meaning a skewed T cell repertoire. There was also low T cell proliferation and undetectable T cells receptor excision circles (TRECs), highlighting lack of T cell maturation. Additionally, B cell lymphopenia with elevated circulating plasmablasts and dysreactive B cells were detected, along with low IgM and IgA levels and high IgE, which are not a typical feature of CDG-related immunodeficiencies, but may explain autoimmunity (**Supplementary Table 1**) (41). There were no investigations directly linking MAN2B2 deficiency to the observed immune alterations. Nevertheless, the intercellular adhesion molecule 1 (ICAM1) adhesion protein and lysosomal associated membrane protein 2 (LAMP2), known to play a critical role in T cell regulation (42, 43), are underglycosylated in the patient’s fibroblasts (41), suggesting the protein functions are affected and deserving further studies to address the exact mechanisms. Yet, a newly reported Chinese patient presented normal immune parameters alongside presentation of several malformations (44), which raises the question if MAN2B2-CDG is as a condition with major immunological involvement or a disorder with infrequent immunological occurrences.

Due to the small number of MAN2B2-CDG patients described, there is a lack of information regarding treatment response. One patient receiving immunoglobulin replacement therapy initiated at two years of age failed to improve the immune manifestations. Yet, hematopoietic stem cell transplantation (HCST) at the age of five years restored T cell count and function, antibodies production and resolved the infection episodes (41).

### 3.2 Combined immunodeficiencies with syndromic or associated features

EXTL3- and PGM3-CDG are the CDG included in the category of combined immunodeficiencies with syndromic features, presenting variable immunophenotypes ranging from isolated infections to severe immunodeficiency, including neurodevelopmental and skeletal defects. EXTL3-CDG compromises the glycosaminoglycan and the heparan sulfate biosynthesis by affecting GlcNAc transfer (**Figure 2**). Alterations in the length and content of heparan sulfate are known to disbalance biological mechanisms and signaling pathways. On the one hand, heparan sulfate has a critical role in the binding and directing cells and growth factors during thymic epithelial cell differentiation and hematopoietic progenitor cell expansion (45–47). Consistently, EXTL3-CDG immunodeficiency was shown to majorly derive from defects in early T cell development (48). On the other hand, heparan sulfate alterations increase fibroblasts growth factor receptor signaling by increasing ligand binding, concordant with previously reported mechanisms of skeletal dysplasia. Besides, decreased STAT5 phosphorylation in response to IL-2 and IL-7 was found in patient immune cells indicative of impaired cellular responses (48). In addition to patients with variable immunophenotypes, some patients (n = 3/16) have no apparent immune issues (49). The cause of EXTL3-CDG clinical variability has been suggested to be related to the associated genetic variants. Variants affecting the EXT domain seem to be related with skeletal defects whilst variants affecting the transferase activity resulting in defective glycosaminoglycans modifications associated with immunophenotypes. However, patients with the same mutation can greatly differ phenotypically (50). Other genetic interactions, gene modifiers or environmental factors are possibly behind the different degrees of immunodeficiency severity (48).

In PGM3-CDG, there is an impairment on the synthesis of UDP-GlcNAc due to the deficient PGM3 activity (**Figure 2**). This deficiency affects the number and function of different immune cells resulting in repeated dermal and pulmonary bacterial or fungal infections (**Supplementary Table 1**). These manifestations seem to mainly derive from altered signaling pathways disbalancing the immune response towards a Th2 profile (51, 52). Specifically, in PGM3-CDG, the N- and O-glycosylation of T cells is impaired. Decreased O-GlcNAcylation disrupts NFAT function (53) and NF-κB transcriptional activation (54) whilst defective N-glycosylation of the gp130 protein impairs STAT3-mediated signaling (55), which negatively interferes with T cell proliferative capacity and cytokine production (53, 55, 56). This results in abnormal levels of various cytokines (e.g., IL-6, IL-17, IL-27, and granulocyte colony stimulating factor - GCSF) which impairs cytokine-mediated immune responses, such as memory cell differentiation, and elevates IgE associated allergy and atopy – a hallmark of PGM3-CDG (55, 57). The dysregulation of the metabolic profile of PGM3-deficient cells was also proposed as a contributing factor to the immune issues (58).

HSCT has been successfully performed as a treatment for EXTL3-CDG and PGM3-CDG immunodeficiency recovering the normal T cell development in these patients and is already approved for PGM3-CDG (59, 60).

### 3.3 Predominantly antibody deficiencies

ALG12-, ATP6AP1-, and MOGS-CDG fall within as the category of predominantly antibody deficiencies since all of them present hypogammaglobulinemia, with low IgG levels. Despite this common immune hallmark, each disorder presents distinct clinical features. ALG12- and MOGS-CDG are multi-systemic disorders with development delays, hypotonia, dysmorphism and various organ-related manifestations, while ATP6AP1-CDG mainly presents an immunodeficient phenotype with liver involvement.

Defects in either ALG12 or MOGS compromise N-glycan biosynthesis (61, 62). ALG12 is a mannosyltransferase responsible for adding the eighth mannose to the lipid-linked oligosaccharide precursor, whereas MOGS is a glucosidase that catalyzes the trimming of the first glucose residue from the Glc3-Man9-GlcNAc2 precursor (**Figure 2**). In turn, ATP6AP1 is the first accessory subunit of the proton transporting vacuolar (V)-ATPase pump (**Figure 2**) whose defects compromises both N-glycosylation and mucin type O-glycosylation (63, 64). This leads to deficient antibody glycosylation that decrease their thermal stability, resistance to unfolding and proteolytic cleavage (65) and interferes with their function by affecting antibody binding to Fc receptors (66, 67). Consequently, this likely contributes to the genesis of the antibody deficiency, inflammatory episodes and absent responses to vaccines observed in ALG12-, MOGS-, and ATP6AP1-CDG. The hypogammaglobulinemia reported in ALG12- and MOGS-CDG patients (68, 69) translates into propensity for recurrent and severe bacterial infections due affected antibody-mediate immune processes. Altered lymphocyte counts and dysfunction are also common even though the causative mechanisms are still not understood (**Supplementary Table 1**). It has been described that MOGS-CDG patients have resistance to glycosylation-dependent enveloped viruses. This can be explained by the fact that these viruses often hijack the host glycosylation machinery to modify their viral proteins required for viral replication and cellular entry (70, 71). Yet, a recent report challenges this hypothesis, by identifying several infections by enveloped viruses in two patients (62, 72–74).

Considering that these CDG belong to the category of inborn error of immunity mainly associated with decreased levels of circulating Igs, some ALG12-CDG patients have been treated with Ig infusions with apparent no success (61, 75). Similar therapeutic strategies to control immune manifestations in MOGS- or ATP6AP1-CDG are not known in the literature. Nevertheless, Ig infusions are widely recommended to this category of inborn error of immunity.

### 3.4 Diseases of immune dysregulation

Magnesium transporter 1 (MAGT1) deficiency has been only recently recognized as a CDG after the unveiling that MAGT1 besides function as Mg^2+^ transporter is a critical accessory protein for immune cell N-glycosylation (76–78). Clinically, most patients present a disease named X-linked MAGT1 deficiency with increased predisposition to Epstein-Barr virus (EBV) infection and N-linked glycosylation defects (XMEN) (76, 78–80), having increased susceptibility to viral respiratory, oral, and skin infections and, in some cases, autoimmune conditions and alterations in vaccination responses (**Supplementary Table 1**). More recently, variations of the MAGT1-CDG phenotype were described with one patient with muscle involvement characterized by myositis with immune infiltrates (81) and two patients with a different neurological phenotype without immune involvement (79). However, the causes of clinical variability are still elusive.

The selective immune dysregulation in MAGT1-CDG with the XMEN phenotype stems from STT3B-dependent glycosylation alterations of immune-related molecules. In fact, MAGT1 is part of the STT3B complex which is a subunit of the OST complex (**Figure 2**). Therefore, even though the exact mechanisms are not yet understood, the Mg^2+^ transport defect results in incomplete glycosylation of a subgroup of STT3B substrate proteins (77, 79). Namely, among others, defective glycosylation of NKG2D, a regulator of natural killer (NK) and CD8^+^ T cells responsiveness, reduces its stability and membrane expression while increasing its degradation, compromising the anti-viral immune responses (78). The T cells co-stimulator CD28 is also hypoglycosylated, impairing CD28-mediated cell signaling required for immune processes, such as leukocyte activation, movement, apoptosis regulation, cellular adhesion, and adaptive immune responses. Importantly, CD70 underexpression and hypoglycosylation was also found, which predisposes individuals to uncontrolled EBV infection by affecting humoral and cell-mediated immunity in humans through CD27-CD70 impaired signaling, especially important for the control of EBV (78, 82). Ig hypoglycosylation was observed, explaining the hypogammaglobulinemia in about half of the reported patients (**Supplementary Table 1**) (77). These observations not only support the association of MAGT1 deficiency with CDG but also explain the increased EBV viremia and a higher risk of EBV-related B cell lymphomas and lymphoproliferative disorders which are associated with increased mortality in these patients (83).

Patients with EBV-related malignancies are managed with radio and/or radiotherapy with varying outcomes (76). The use of magnesium supplementation in MAGT1-CDG patients with an XMEN phenotype showed mixed results. While it partially recovered NKG2D expression and reduced persistent EBV infections and likely reduced EBV-associated malignancies in some patients (84), others did not respond to supplementation (85). Alternative therapeutic approaches have been tested. One patient was treated with a MAGT1 messenger RNA with restoration of NKG2D expression and CD8^+^ T and NK cell levels (86). More recently, an *ex vivo* approach using CRISPR/Cas9 adeno-associated vector able to insert a *MAGT1* gene in T lymphocytes or hematopoietic stem and progenitor cells from MAGT1-CDG patients was optimised which recoved NKG2D expression and function in NK and CD8^+^ T cells in an immunodeficient mice (87). These studies bring some hope in developing effective therapeutic options for this CDG.

### 3.5 Congenital defects of phagocyte number, function, or both

SLC35C1-, G6PC3-, JAGN1-, and VPS13B-CDG are diseases related to phagocytic cells which, subsequently, manifest with frequent and severe infections, particularly affecting the skin, oral cavity, and respiratory tract (**Supplementary Table 1**).

SLC35C1 codes for a transporter of GDP-fucose (**Figure 2**) and *SLC35C1* pathogenic variants affect the biosynthesis of selectin ligands and other fucosylated glycoproteins important for leukocyte adhesion (88, 89). Thus, SLC35C1-CDG, also called Leukocyte Adhesion Deficiency type II (LAD-II), shows impairment of leukocyte migration and homing due to decreased expression of selectin ligands, with subsequent propensity for infections (90). The clinical phenotype includes milder intellectual disability, short stature (91–93), and, in severe cases dysmorphia, immunodeficiency, and the Bombay blood type (94, 95). Oral fucose supplementation was found to not only improve the neurological development of SLC35C1-CDG patients, but also reduce the number of recurrent infections and normalize the neutrophil counts. However, fucose supplementation can induce autoimmunity, as the appearance of fucosylated neoantigens on the cells may induce the production of autoantibodies (94, 96).

Genetic variants in *G6PC3*, *JAGN1*, and *VPS13B* affect the number and function of neutrophils (rather than their migration) which consequently cause different forms of severe congenital neutropenia (SCN). While G6PC3- and JAGN1-CDG show persistent neutropenia and bone marrow defects, VPS13B-CDG presents intermittent decreased neutropenia with normal bone marrow development and cellularity (**Supplementary Table 1**). Besides, G6PC3-CDG and VPS13B-CDG patients present Dursun and Cohen syndrome, respectively, whereas JAGN1-CDG presents solely SCN (97, 98). These phenotypes are likely to arise from the distinct roles of these enzymes in the glycosylation process. Specifically, G6PC3 catalyzes the last step of glycogenolysis in the ER (**Figure 2**), decreasing the levels of glucose and glucose-6-P in the cytoplasm. Additionally, G6PC3-CDG patient neutrophils show truncated, galactose-defective N- and O-glycans (99). JAGN1 is involved in the vesicle-mediated transport in the glycosylation pathway (**Figure 2**) and its deficiency leads to increased Gal-α-1,3-Gal terminated triantennary glycans and decreased sialylated biantennary glycans (100). VPS13B protein mediates lipid transfer between membranes (**Figure 2**) and defects in this protein causes Golgi disorganization, hypoglycosylation of early endosome antigen 1 and of lysosome-associated membrane glycoprotein 2, as well as N-glycan maturation defects (101–103).

Even though the direct cause of glycosylation-related pathological effects is still elusive, proposed mechanisms for the multi-factorial neutropenic phenotypes, include increased neutrophil death associated with metabolic dysregulation, ER stress and apoptosis, and decreased levels of respiratory burst, calcium mobilization, and neutrophil survival factor (SERPINB1) (103–106); neutrophil dysfunction with abnormal protein glycosylation impacting neutrophil migration, adhesion and cytotoxicity (101, 105, 107); and bone marrow maturation defects and in some cases myelokathexis (99, 108, 109). Consequently, these defects impact the ability of neutrophils to correctly fight infections.

Besides neutrophils, other immune cells and antibodies can be altered in the CDG included in this category (**Supplementary Table 1**). In fact, abnormal B cell counts, and function were detected, associated with altered ER homeostasis and aberrant Ig N-glycosylation in a *Jagn1* deficient mouse model and patient-derived cells (110). Inflammatory and autoimmune manifestations, especially in G6PC3-CDG include IBD with 16% of prevalence (**Supplementary Table 1**). It was suggested that IBD prevalence in this CDG could derive from higher and consistent neutrophil activation leading to elevated expression of adhesion molecules, inflammatory cytokines, and reactive oxygen species (111).

Treatment with sodium-glucose cotransporter 2 inhibitors, which inhibit renal glucose reabsorption, improved neutrophil counts and function and reduced the severity of infection episodes in G6PC3-CDG patients (112, 113). On the other hand, GCSF is typically used in neutropenia management but failed to show therapeutic efficacy, despite increasing neutrophil production (112). GCSF treatment also showed poor response when used in patients with *JAGN1* mutations and HSCT has been the treatment of choice for JAGN1-CDG (114, 115). Regarding VPS13B-CDG, GCSF can be used to control the neutrophil levels (116, 117).

### 3.6 FUT8-CDG: predominantly lung immune dysfunction

Although FUT8-CDG has not been yet categorized as an inborn error of immunity, all described patients show strong immunological involvement especially in the lung. FUT8-CDG is caused by genetic variants in fucosyltransferase 8 (FUT8) which catalyzes the transfer of the fucose residue from GPD-fucose to the first GlcNAc residue of N-glycans, known as core fucosylation (**Figure 2**). Presently, only nine known FUT8-CDG patients exhibit severe multi-organ disease (118–120) with infection and inflammatory events of the lungs and respiratory tract. These immune manifestations are mostly recurrent leading to respiratory failure in severe instances (**Supplementary Table 1**) (120). The reason for the variable outcomes in FUT8-CDG is still elusive. The lung immune dysfunction observed in FUT8-CDG is likely related to dysregulation of the TGF-β1 receptor. Specifically, studies in *Fut8* ^-/-^ mice show that defects in TGF1-β1 activation and signaling, leading to overexpression of matrix metalloproteinases and downregulation of extracellular matrix proteins, resulting in delayed alveolar epithelial differentiation and the presence of emphysema-like lung abnormalities (121).

Cellularly and biochemically, the few available reports show the occurrence of neutropenia and IgG hypofucosylation in FUT8-CDG. Nevertheless, FUT8-related depletion of core fucosylation has been linked to antibody dysfunction, profound alterations in B and T cells, hypogammaglobulinemia, as well as impaired recognition, assembly, and lipid raft association of pre- and IgG-B cell receptor antigen (122–127). Even though these finding underline a key role of FUT8 in controlling lung immunity and inflammation, further detailed investigations are warranted to: (1) unveil systemic immune response impact, which might become apparent as more patients are diagnosed, and (2) accurately categorize FUT8-CDG as an inborn error of immunity.

Fucose supplementation has been explored for FUT8-CDG with mild improvement of the clinical phenotype in two patients. However, a more noticeable effect may require adjusting the amount of fucose (118). In case of severe respiratory disease, supportive measures like continuous positive airway pressure and tracheostomy have been adopted (118, 120).

## 4 PMM2-CDG: a CDG with minor immunological involvement

**Figure 3:**
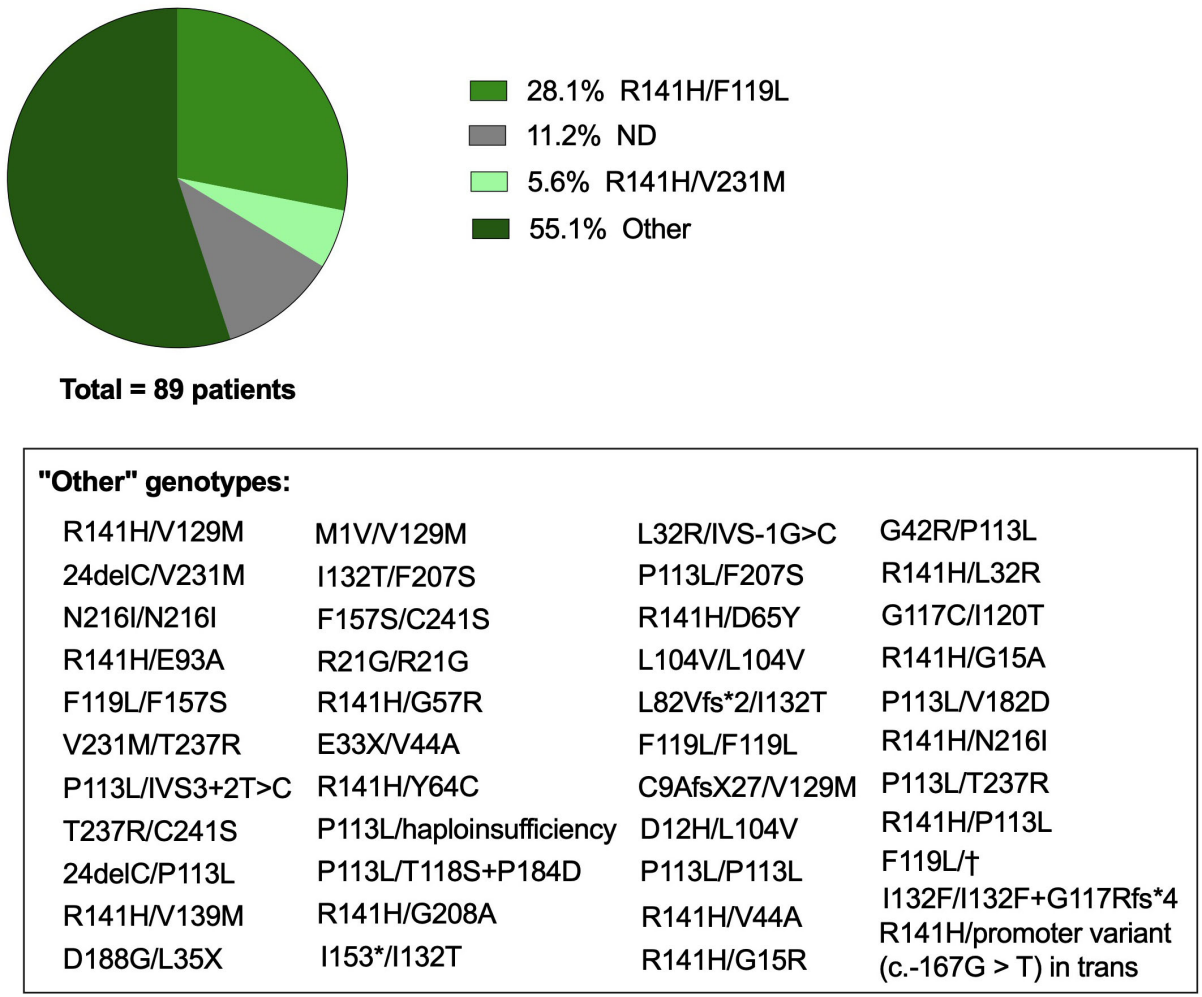
Genotype of PMM2-CDG patients with immune involvement. Genetic background of PMM2-CDG patients with immune involvement captured following a literature revision. Articles were captured through an automated python search through the MEDLINE database and using PubMed as the search engine. The list of keywords, inclusion and exclusion criteria, selected articles and data collection are described in **Supplementary File 1**. The category ‘Other’ includes genotypes that appeared in less than 5% of the patients, representing in total less than 3 reported patients per genotype, which are described in the text box. ND, Not defined or not reported; †, intronic mutation c.639-1G>T. Created with GraphPad Prism 8.

While this revision provides an in-depth analysis of the twelve CDG with prominent immunological features, it is important to note that other CDG also present immune issues, though less frequent yet not always less severe. A notable example is phosphomannomutase 2 (PMM2)-CDG (MIM: 212065), the most common CDG, with an estimated incidence of 1:20,000 people, and the first ever described CDG in 1980 (128, 129). PMM2-CDG is caused by pathogenic autosomal recessive genetic variants in *PMM2* which affects PMM2 activity. PMM2 catalyzes the conversion of mannose-6-phosphate into mannose-1-phosphate, a precursor of guanosine diphosphate mannose (GDP-Man) and dolichol-P-mannose (Dol-P-Man) required for the precursor oligosaccharide assembly during N-glycosylation (**Figure 2**). PMM2 deficiency leads to hypoglycosylation of numerous glycoproteins, causing multi-organ involvement and broad-spectrum presentations, ranging from severe neonatal to mild adulthood presentation. Besides the major defective neurological phenotype, several immunological complications and abnormalities have been reported in PMM2-CDG patients over the years. To have a comprehensive understanding of the immune dysfunction in PMM2-CDG, we performed a literature revision using an automated python search through the MEDLINE database and using PubMed as the search engine (**Supplementary File 1**). Based on the literature revision, 89 patients were reported to present clinical manifestations related to the immune system. In this cohort, 46 distinct genotypes were identified, among which 41 are heterozygotic and 5 are homozygotic (**Figure 3**). Almost a third of these patients (n = 25/89, 28.1%) present the R141H/F119L genotype and 5.6% (n = 5/89) the R141H/V231M genotype. Other genotypes associated with immune issues occur in the remaining 55.1% of the patients and account for three or less patients per genotype (**Figure 3**). Notably, there are two prevalent heterozygous genetic variants – R141H and F119L – which are present in 50 (56.2%) and 29 (32.6%) patients, respectively.

While PMM2-CDG is not predominantly known for its immune complications, and such issues mainly prevail during infancy, the impact when they do occur can be profound (**Table 1**). Infection was the most reported event, with approximately half of the patients experiencing at least one infection episode. This can lead to severe outcomes, including sepsis, septic shock, multi-organ failure and/or fatalities. At the time these reports, 19 patients (21.3%) from this cohort had succumbed to complications due to immunological issues or other conditions. Specifically, 10 (11.2%) of these patients passed away due to pneumonia, unidentified infections, rotavirus, and adenovirus. However, not all infections led to extremely dire outcomes, some being mild while others also resulting in hospitalizations for conditions like Influenza, enterovirus, and Sars-CoV-2. These episodes often triggered other clinical manifestations, mainly neurological like seizures and stroke-like episodes, as previously reported (168). Other immune manifestations, such as gastric and bowel inflammation, vasculitis, pericarditis, and eczema, as well as immune effector alterations like hypogammaglobulinemia and altered white blood counts, have also been less frequently described in PMM2-CDG patients. Notably, one patient succumbed to respiratory insufficiency and heart failure following vaccination.

**Table 1:** Clinical features and biochemical parameters of reported PMM2-CDG patients with immunological involvement.

Number of patients with immune involvement	Genetic variants (Va/Vb)	Age (at report)	Clinical Features		Biochemical Parameters	Observations	Reference
			Infections (pathogen, when identified)	Immune manifestations			
**1**	R141H/V129M	2 y 6 m		Recurrent episodes of fever related to hypogammaglobulinemia	Hypogammaglobulinemia	Required Ig infusions	(130)
**21**	R141H/F119L	0–19 y (mean 7.6 y)	Some presented intercurrent infections	Febrile seizures		Two died of pneumonia (4.3 and 6 y), one with septic shock (1.8 y), one with unclassified infection (9.9 y); twelve had SLE, often associated with fever and seizures	(131)
**1**	24delC/V231M	10 m	Three septic-like events (0–2m, inconclusive bacterial infection)	High fever	↓ CRP; leukocytosis	Serum transaminase concentrations highly increased during the septic like-events	(132)
**6**	R141H/-	–	Infection episodes	Fever (during infections)		Infection episodes associated with seizures	(133)
**1**	N216I/N216I	16 m	Several upper airways infections; bronchopneumonia (4 m)				(134)
**2**	–		–		Hypogammaglobulinemia		(135)
**1**	–	3 m	Neonatal sepsis (*S. aureus*)	Persistent dermatitis			(136)
**1**	R141H/E93A/	6 y	Upper UTI and frequent RTI until 2 y		Leukocytosis with significant left shift		(137)
**1**	F119L/F157S	Deceased		Macrophage activation, phagocytosis of erythropoietic elements (newborn)		Died at 8 w from deterioration of respiratory and myocardial function	(138)
**1**	V231M/T237R	Deceased	Pneumonia (1 w, *Klebsiella*)			Died of circulatory and respiratory failure at 1 m	(139)
**2**	P113L/IVS3 + 2T>C	6 y	Recurrent otitis media (< 1 y); pneumonia (including *Pseudomonas*); *Pseudomonas* cellulitis; chronic mucocutaneous *Candida*; recurrent line sepsis	↓ neutrophil chemotaxis; poor vaccination response	Leukocytosis; leukopenia; ↑ CD19^+^; hypogammaglobulinemia	IVIG treatment	(140)
	P113L/IVS3 + 2T>C	4 y	Recurrent otitis media (< 1 y); pneumonia (RSV and *Influenza*); chronic mucocutaneous *Candida*; recurrent line sepsis; *E. coli* sepsis; *S. viridans* endocarditis	Poor vaccination response; fever and vesicles post varicella vaccination; delayed-type hypersensitivity anergic to mumps and *Candida*	Leukocytosis; CD19^+^, hypogammaglobulinemia	IVIG treatment	
**1**	T237R/C241S	5 y		Intraepithelial lymphocyte infiltration (18 m)			(141)
**1**	F119L/F157S	Deceased	Recurrent infections (bacteria)		Hypogammaglobulinemia	Died at 8 w due to severe failure to thrive and recurrent infections	(142)
**2**	24delC/P113L	6 y	–	Diffused cerebral vasculitis			(143)
	R141H/V139M	8 y	–	Pericarditis			
**1**	–	6 y	Neonatal sepsis; gastroenteritis (2 m); recurrent upper RTI		Progressive lymphopenia	Cow milk intolerance	(144)
**2**	R141H/F119L	Deceased	Adenovirus infection			Diet at 6 y due to acute ascites and pericardial effusion during infection;	(145)
	D188G/L35X	Deceased	Minor infections with recurrent hypoalbuminemia, ascites, tachypnea, and dyspnea			Died at 1.8 y (ascites and pericardial effusion in multi-organ failure)	
**1**	M1V/V129M	Deceased	Lung infection at 3 m			Lung infection was fatal	(146)
**2**	I132T/F207S	10 w	Few infections and bronchiolitis (viral)				(147)
	R141H/V231M	5 y	–	Necrotizing enterocolitis (neonatal)			
**1**	F157S/C241S	4 y 6 m	Chickenpox (viral)				(148)
**2**	R141H/F119L	12 y	Group A *Streptococcus*, *E. coli* (bacterial infections)			Worsening of lymph vessel structure due to infections	(149)
	R21G/R21G	29 y	Recurrent infections (bacterial; 2 m)				
**1**	R141H/G57R	3.5 m			Leukocytosis		(150)
**5**	E33X/V44A	1 y	Severe viral infections (rotavirus at 3 m, complicated with post-natal CMV infection)		↓ CD3^+^; ↑ CD16^+^/CD3+ ratio	Rotavirus infection required ICU admission for 2 m	(151)
	R141H/V231M	Deceased	Severe viral infections at 5.5 m (adenovirus) and at 1 y (rotavirus)		↓ CD3; ↑ CD16^+^; ↑ CD16^+^/CD3+ ratio	Died at 14 m (multi-organ failure) after ICU admission due to infection	
	R141H/Y64C	4 y	Severe viral infections: enteritis at 5 m (rotavirus) and 8 m (adenovirus), bronchiolitis (RSV) at 5 m; recurrent ear infections		↓ CD3	Ear infections were responsive to oral antibiotics	
	P113L/ haploinsufficiency	4 y			↓ CD16^+^		
	P113L/T118S+P184D	9 y			↓ CD3		
**2**	R141H/V231M	Deceased	Pneumonia (21 m)		T lymphopenia (↓ CD3, CD4 and CD8); hypogammaglobulinemia (IgG and IgM)	Died at 21 m due to heart failure and pulmonary edema (fatal infection crisis)	(152)
	R141H/G208A	Deceased	Episodes of bacterial sepsis (1 y)			Died at 2 y (unknown cause of death)	
**1**	I153*/I132T	8 m	Recurrent lower respiratory infections (1/month at 3 m and 3/month at 3 y)		Persistent low CD4^+^ T cells hypogammaglobulinemia (IgG)	IVIG treatment	(153)
**3**	L32R/IVS-1G>C	14 y 5 m	Enterovirus			Infection was a SLE trigger	(154)
	P113L/F207S	3 y 3 m	Influenza virus infection			Infection was a SLE trigger	
	R141H/D65Y	5 y 8 m	Upper viral RTI			Infection was a SLE trigger	
**1**	L104V/L104V	6 y			Persistent leukocytosis (from 6 to 9 y)		(155)
**1**	R141H/V231M	Deceased	Two infection episodes at 10 m (*A. baumanii* detected in one episode)	Fever (during infections)	Leukocytosis (6 w)	Died at 11 m from respiratory insufficiency and heart failure after vaccination	(156)
**1**	L82Vfs*2/I132T	7 y 6 m		Febrile seizures			(157)
**1**	I132F/I132F+G117Rfs*	Deceased	Presumably viral encephalitis (6 w)			Died at 10 m (cardiac issues)	(158)
**1**	F119L/F119L	21 y	Several infections (5 y)	Febrile seizures (4 y)			(159)
**1**	C9Afs*27/V129M	Deceased			Pancytopenia	Unknown cause of death	(160)
**1**	D12H/L104V	23 y	Common cold (11 y)			Common cold lead to hospital visit	(161)
**2**	P113L/P113L	13 y	Gastroenteritis (viral)	Refractory fever			(162)
	R141H/V44A	24 y	Several unexplicit infections (see observation)			Infection with associated hyperthermia and epileptic seizures triggered somnolence and irritability	
**2**	R141H/G15R	Deceased	Recurrent infections); gastroenteritis (*C. difficile* 7 m, Parvovirus 46 m); sinusitis (46 m)	Eczema; febrile illness	Pancytopenia	Died at 59 m at the hospital (admitted due to pain)	(163)
	G42R/P113L	Deceased	Recurrent infections; pneumonia and sepsis (9 y)			Died at 11 y and 6 m due to end stage renal disease	
**1**	R141H/L32R	13 y	Recurrent RTI (3 y); pneumonia (4 y)				(164)
**1**	G117C/I120T	2 m	Pneumonia				(165)
**3**	R141H/promoter variant (c.-167G > T)	6 y		Eczema; GI inflammation; colon moderate pancolitis, cryptitis			(166)
	R141H/promoter variant (c.-167G > T)	10 y		Active gastric inflammation; pancolitis with cryptitis			
	R141H/promoter variant (c.-167G > T)*/*	6 y		Eczema; eosinophilic esophagitis; gastritis; inflammation with cryptitis; egg anaphylaxis			
**10**	R141H/G15A/	3 y	Unspecified infection			DVT simultaneous with infection	(167)
	P113L/V182D	7 y		Febrile illness		Developed SLE during febrile illness	
	T237R/C241S	8 y		Febrile illness		Developed SLE during febrile illness	
	R141H/N216I	3 y	Septic episode (9 m)			Septic episode with coagulopathy resulting in DVT (9 m)	
	P113L/T237R	7 y		High fever		Fever associated with SLE	
	R141H/F119L	29 y	SARS-CoV-2 infection	Febrile illness		SARS-CoV-2 infection led to hospitalization. Developed SLE during febrile illness	
	R141H/V231M	Deceased		Febrile illness		Developed SLE during febrile illness. Died at 12 m from liver failure and portal venous thrombosis.	
	R141H/F119L	1 y	Possible sepsis				
	R141H/P113L	9 y		Febrile illness		Developed SLE during febrile illness	
	F119L/†	26 y		Periods of fever		Fever associated with SLE (> 17 y)	

Clinical and immunological data represented were obtained following a literature revision using an automated python search through the MEDLINE database and using PubMed as the search engine (detailed in **Supplementary File 1**).

a CD, cluster of differentiation; CRP, C reactive protein; CMV, Cytomegalovirus; DVT, deep venous thrombosis; ICU, intensive care unit; Ig, immunoglobulin; IVIG, intravenous immunoglobulin; m, months old; RSV, respiratory syncytial virus; RTI, respiratory tract infections; SLE, stroke-like episodes; UTI, urinary tract infection; Va, variant a; Vb, variant b; WBC, white blood cells; y, years (old); m, months (old); w, weeks (old). † - intronic mutation c.639-1G>T.

Up arrow - increased or high.

Low arrow - decreased or low.

### 4.1 PMM2-CDG glycan profile

PMM2-CDG leads to profound alterations in N-glycosylation, resulting in incomplete glycan chains and truncated lipid-linked oligosaccharides (169). Serum protein analysis reveals decreased tetrasialotransferrin and increased disialotransferrin and asialylated isoforms, aiding PMM2-CDG screening (34). Besides transferrin, many liver proteins show N-glycan defects which contribute a range of symptoms and complications. While few studies explore patient-derived cells, altered mannosylation and mannose-terminal glycans are evident. Despite the reduced mannose incorporation in fibroblasts from PMM2-CDG patients (169), an intriguing report showcased hypermannosylation of monocytes in two patients (170). The intricate landscape of oligomannosidic glycans showed a reduction in long glycans (171), but an increase in short high mannose glycans, such as Man3GlcNAc2 and Man4GlcNA2 in several patients (171–173).

Other glycan alterations include low levels of a non-mannosylated tetrasaccharide - Neu5Acα2,6Galβ1,4-GlcNAcβ1,4GlcNAc (171, 172) and elevation of endogenous glycosphingolipids in patient fibroblasts (174). Aberrant sialylation is observed in PMM2-CDG patients. Particularly, low sialylation was observed in platelets, fibroblasts, and B cells of patients (174–177), contrasting with high sialylation seen in monocytes of three patients (170).

Overall, PMM2-CDG patients have a distinctive glycan profile characterized by inferior number of N-glycosylation sites, incomplete N-glycans with terminal mannose residues, reduced sialylation, and increased expression of glycosphingolipids. This multifaced glycan profile contributes to molecular interaction and cell recognition, underpinning the disease’s symptoms and complications.

### 4.2 Altered glycosylation could explain immune involvement in PMM2-CDG

The altered glycan profile of PMM2-CDG patients could be directly or indirectly implicated in the immunological manifestations reported in these patients (**Table 1**). Firstly, compromised glycosylation indicates a disruption in the recognition and response triggering of ligands by immune receptors. For example, glycosylation of Toll-like receptors and other pathogen recognition receptors is pivotal in the recognition and binding of pathogen structural components in these heavily glycosylated structures, affecting and conditioning the optimal functioning of these receptors (178–182). This means that, in a disease as PMM2-CDG, where hypoglycosylation is prevalent, the impaired general glycosylation caused by this defect early in the N-glycosylation pathway could compromise the effective recognition of threats by immune receptors and the overall immunological response in these patients, leading to a possibly higher susceptibility to recurrent and severe infections in PMM2-CDG patients.

Moreover, increased truncated surface N-glycans have been associated with chronic gastrointestinal inflammation, such as IBD (183). One of the mechanisms known to be associated with IBD and other autoimmune diseases severity is a deficiency in branched N-glycans in the mucosal T lymphocytes, which has been associated with mutations in the *MGAT5* gene, leading to a hyperimmune response (184, 185) With a mutation in the PMM2 enzyme, an early defect in the N-glycosylation pathway also leading to truncated N-glycans, a decrease in complex and branched glycans, and a known heterogenous clinical phenotype in PMM2-CDG, it is possible that the gastrointestinal inflammatory events observed in patients share the same mechanisms as IBD and other autoimmune diseases. The study of mannose in the regulation of immune processes showed that increased levels of mannosylated glycans might play a role in inducing inflammatory autoimmune-type responses, such as in systemic lupus erythematosus (SLE) (186). The higher abundance of mannosylated glycans has been associated with SLE through increased γδ T lymphocyte development and infiltration into the kidneys (187, 188). Additionally, the alteration of terminal sialic acid levels observed in PMM2-CDG might point towards the dysregulation of inflammation and autoimmunity processes (189, 190). In fact, hyposialylation of immunoglobulins has been linked to autoimmune pathologies, such as granulomatosis, chronic inflammatory demyelinating polyneuropathy and rheumatoid arthritis (189). Besides, sialic acid has been shown to have an important role in regulating the activation, proliferation, modulation, and orientation of several immune cells, including dendritic cells, T and B cells (189, 191). Although these observations may contribute to the understanding of infections, inflammatory manifestations in tissues, or even unexplained febrile illness that occurs in PMM2-CDG, caution should be taken while drawing definitive conclusions due to glycan heterogeneity between patients (170).

### 4.3 Possible links between PMM2-CDG glycan profile and susceptibility to infection

The nuanced interplay between host and pathogen constitutes a fundamental facet of the infection. Glycans are known pivotal mediators for pathogen recognition and the cell glycoprofile serves as critical determinants of infection outcomes. The glycans expressed in some PMM2-CDG patient derived tissues have been elucidated, and described above, which allows us to have a general overview of the PMM2-CDG glycan profile.

Altered glycans in the PMM2-CDG host may also indicate higher susceptibility to binding and invasion by microorganisms across diverse cells and tissues, given the existence of a thinner and less dense glycocalyx - a glycan-rich structure which protects cells and tissues from aggression (192). The disbalance of the glycocalyx exposes novel glycan epitopes, such as altered patterns of sialylation and exposed mannose, thereby providing pathogens with potential attachment and invasion sites (192).

**Figure 4:**
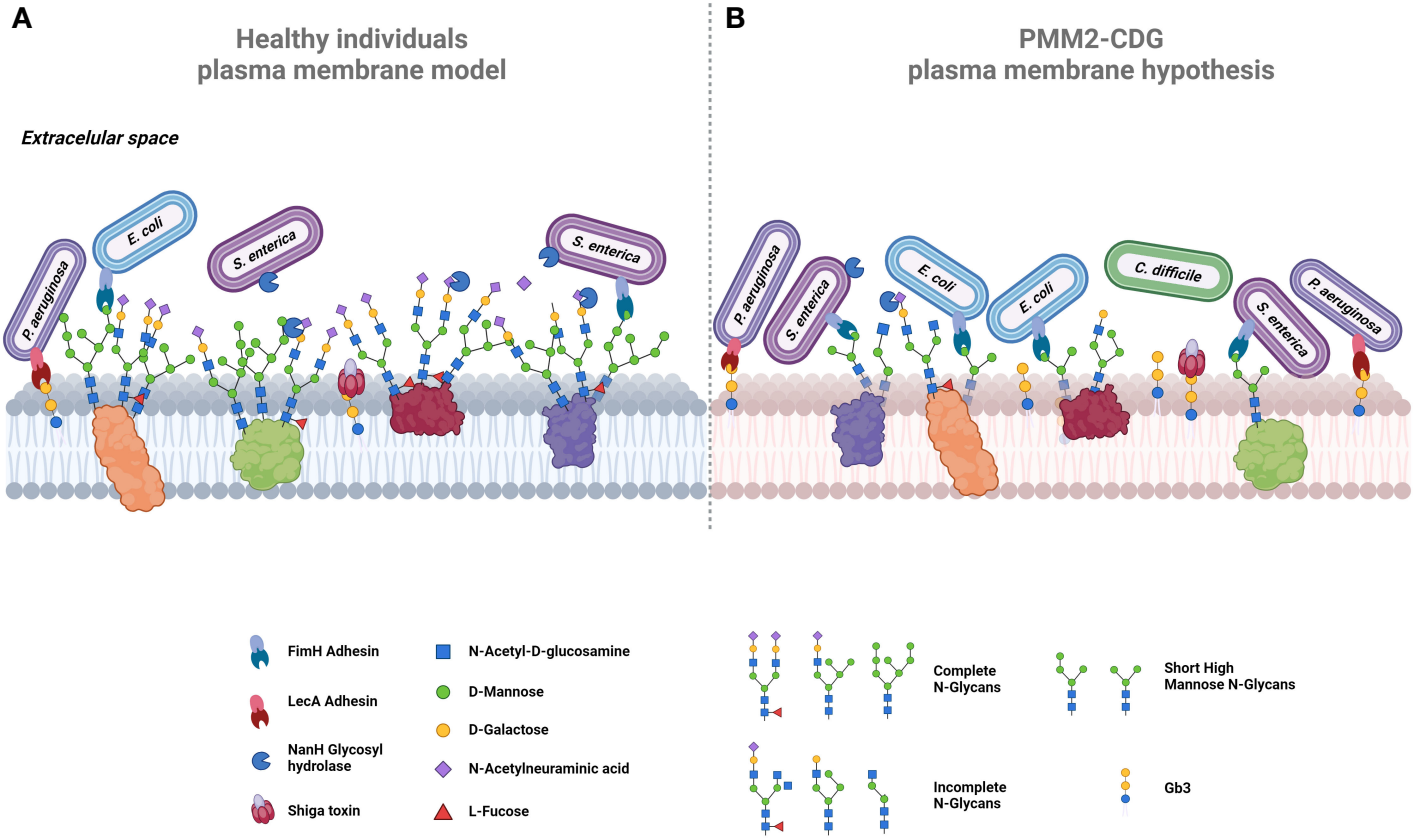
Proposed comparative models of plasma membrane glycosylation. **(A)** In healthy cells, the plasma membrane exhibits glycoproteins decorated by extended N-glycans, collectively forming the glycocalyx. Terminal mannose residues on glycoproteins serve as targets for adhesins, exemplified by FimH in bacteria like *E*. *coli* and *S. enterica*. Certain bacteria, such as *S. enterica*, release glycosyl hydrolases like NamH, which degrade terminal glycans as sialic acid, thereby increasing available binding sites and promoting bacterial adherence. The host glycosphingolipids, including Gb3, also serve as binding sites for adhesins, e.g., LecA in *P. aeruginosa* or for bacterial toxins, such as Shiga toxin. **(B)** In PMM2-CDG cells, aberrant glycosylation could promote bacterial colonization and infection by pathogens such as *C*. *difficile*. Hyposialylation could lead to the exposure of more adhesion sites. Increased short high mannose glycans offer additional binding sites for bacteria expressing FimH (e.g., *E*. *coli* and *S. enterica*), enhancing their invasion. Similarly, elevated Gb3 expression increases Shiga toxin checkpoints, intensifying its lethality. Created using BioRender.com.

**Figure 4** illustrates a comparative analysis between the N-glycosylated plasma membrane of a healthy individual, with the hypothetical model of a PMM2-CDG patient plasma membrane, constructed based on the known main characteristics of these patients’ glycan profile. Within this context, we highlight some potential host-pathogen interactions, where microorganisms infecting PMM2-CDG patients gain advantages by recognizing specific glycan structures or having facilitated access to otherwise protected sites.

Some PMM2-CDG patients exhibit increased synthesis of several glycosphingolipids (174), notably globotriaosylceramide (Gb3), a sphingolipid that serves as the canonical receptor of Shiga toxin, which is a very potent bacterial toxin that can lead to hemolytic and uremic syndrome and, in most severe cases, death (193, 194). This toxin is produced by *Shigella dysenteriae* and by Shiga toxin producing *E. coli*, such as *E. coli* O157:H7 (193). Gb3 also acts as the binding site for lipid-binding adhesins, such as PapG (*E. coli*), LecA (*Pseudomonas aeruginosa*) and SadP (*Streptococcus suis*) (195–197). All these adhesins and Shiga toxin are identified as mediators in the pathogenic infection process. Thus, the exposure of Gb3 in PMM2-CDG patients can increase susceptibility to infection by these pathogenic bacteria through these intermediaries, as illustrated in **Figure 4**. An increased expression of glycosphingolipids, especially Gb3, in PMM2-CDG patients, might render them not only more susceptible to a broader range of infections but also make organs and tissues, typically less affected by microorganisms’ infection, more vulnerable.

Sialic acid, known to facilitate infection by many viruses and bacteria, such as Influenza viruses or *Haemophilus influenza* bacteria, also serves as an energy source for commensal microorganisms (198, 199). Abnormal sialylation can eventually disrupt the microbiota balance, creating a more favorable environment for growth and persistence of pathogenic microorganisms. This imbalance can grant direct access to the host epithelium or availability of secreted products, with *Clostridium difficile* being the bacterial infection mostly associated with gut dysbiosis (**Figure 4**) (200, 201). Additionally, some bacteria have developed strategies to eliminate sialic acid residues to bypass its protective roles within the intricate glycocalyx network. Employing this strategy, the bacteria uncover novel adhesin binding sites and gain closer proximity to the cell membrane to infect the host. Notable examples include glycosyl-hydrolases NanA of *Streptococcus pneumoniae*, and NanH (**Figure 4**) and ChiA of *Salmonella typhimurium*, which degrade terminal sialic acid residues (202–204). In the case of PMM2-CDG patients who present lower levels of sialylation, pathogenic bacteria may find the infection process easier because they do not need to use their neuraminidase to access internal glycan epitopes to initiate their adhesion process. Therefore, the abnormal sialic acid profile in PMM2-CDG might contribute and indicate a disrupted microbiota, creating a conducive environment for pathogenic infections.

The reduction of longer-high mannose glycans and high abundance of short high-mannose glycans observed in PMM2-CDG may also have implication in the recognition by pathogen’s critical adhesion factors needed for the colonization and infection. In **Table 2**, we present several adhesion factors that are known to be mannose sensitive. FimH, a well-studied lectin-like mannose-binding adhesins, is expressed in many bacteria of the *Enterobacteriaceae* family, such as *Escherichia coli*, *Salmonella enterica* and *Klebsiella pneumoniae* (212–215). Specifically, FimH from *E. coli* has a striking and versatile binding capability interacting with a wide range mannose–containing glycans, exhibiting a higher affinity for oligomannose-3 and oligomannose-5 structures with Manα-(1,3) branches (216). These specific glycans, such as Man3GlcNAc2, appear to be more prevalent in PMM2-CDG patients (**Figure 4**). Another mannose-related adhesion factor is CsuA pilus of *Acinetobacter baumannii* which has been confirmed to contribute to biofilm formation and adherence to the respiratory epithelium (211). In summary, an aberrant glycosylated environment with an increased prevalence of terminal mannose-containing glycans can significantly benefit these microorganisms, by providing binding sites in close proximity to the cell surface and granting an advantage over host defenses.

**Table 2:** Adhesins from microorganisms that target glycans modified in PMM2-CDG patients.

Organism	Adhesin	Glycan-specificity	Reference
***Escherichia coli*** ***Salmonella enterica*** ***Klebsiella pneumoniae*** ***Proteus mirabilis***	FimH	Mannose	(205)
***Enterobacter cloacae***	Type 1 Pili	Mannose Man9(GlcNAc)2 oligosaccharides (high affinity)	(206)
***Lactobacillus plantarum***	Msa	Mannose	(207–209)
***Pseudomonas aeruginosa***	LecB	D-α-mannose (Low affinity) L-α-fucose-binding PA-IIL (High affinity)	(210)
**Uropathogenic *E. coli* strains**	PapG	Forsmann antigen (Gb5), globotetraosylceramide (Gb4) and globotriaosylceramide (Gb3)	(25)
***Acinetobacter baumannii***	CsuA pilus	D-mannose	(211)

Although these host glycosylation-based host-pathogen interactions are recognized as contributors to infection, discerning the exact impact of the glycosidic alterations in PMM2-CDG patients on their susceptibility to infections remains challenging. More information on the glycan profiling of patients’ epithelial tissues would provide more accurate information to formulate target research questions and exploratory studies based on available data concerning microorganisms infecting PMM2-CDG patients.

## 5 Discussion, conclusions, and future perspectives

As in most rare diseases, the nature of the pathological mechanisms and specificities of each CDG are still elusive. However, recent advancements in diagnosis and research are progressively unveiling the intricacies of the pathomechanisms, particularly in CDG immunopathology, increasingly recognized over the years (37, 38).

Our review not only updates the clinical picture of CDG with immune involvement, but also categorizes them according to their immunodeficiency profile, as established by the IUIS (39). To this date, 11 CDG have been classified as inborn errors of immunity by the IUIS and we propose the addition of FUT8-CDG to this group, due to the predominance of lung immune dysfunction (120). Nevertheless, classifying CDG is challenging especially when, so few patients are diagnosed and present heterogeneous phenotypes. The case of MAN2B2-CDG exemplifies this challenge, where an initial immunocompromised presentation contrasted with a subsequent case lacking apparent immune dysfunction, highlighting the need for caution in categorization (41, 44). Despite the challenges, the classification of CDG based on their immunodeficiency is crucial. It not only enhances our understanding and clinical management of these diseases but also facilitates a structured analysis of underlying genetic defects, the identification of specific clinical phenotypes, and the potential inclusion of these CDG for targeted treatments tailored to the unique needs of each immunodeficiency.

Our work provides, for the first time, an exhaustive review of PMM2-CDG patients with immunological involvement. Our comprehensive literature review shows that immunological events in PMM2-CDG, mostly represented as increased susceptibility to infection, are prevalent during infancy and childhood and occasionally lead to severe, sometimes fatal, outcomes. Our review data highlighted that immunologically affected patients predominantly have R141H-bearing genotypes, in line with previous reports (128, 168) and probably because the R141H genetic variant is the most prevalent pathogenic *PMM2* variant (30, 217). Yet, the limited evidence per genotype hampers the establishment of genotype-phenotype correlations. Of note is the fact that we identified 45 genotypes related to immune involvement in PMM2-CDG patients. Nevertheless, 28 additional genotypes captured through patient-reported data (168), highlight the need for comprehensive studies on immune-related clinical events to fill existing gaps. Moreover, while pinpointing precise genotyping correlations remains challenging, the pattern of immune involvement observed in PMM2-CDG contrasts with the more consistent immune deficiencies seen in other CDG, where abnormal immune cell development or function often serves as a defining feature. This contrast underlines the critical yet variable nature of immune involvement across different CDG. Such variability emphasizes the importance of recognizing and managing the broad spectrum of immune dysfunctions in CDG, including the occasionally severe complications encountered in CDG like PMM2-CDG.

This review highlights the broader implications of glycosylation defects, showing that the changes in the glycosylation inherent to CDG genetic defects result can significantly dictate the patient’s immunophenotype. They are associated with dysregulation and the inability to trigger an adequate and balanced immune response which is justifiable by a plethora of dysregulated immune parameters, such as immune cells and antibodies, whose function is inevitably modulated by glycans. Besides, we hypothesize that altered glycan profiles create microenvironments that favor infection, by promoting adherence and colonization by specific pathogens. Taking the example of PMM2-CDG, we established associations between the altered glycan profile and reported clinical immunological manifestations and we suggest potential host-pathogen interactions. Nevertheless, there are some limitations that difficult the establishment of such relationships, as follows:

(a) The gaps in knowledge on CDG immunophenotypes arise from the lack of methodical investigation of immunological parameters between health centers and from the lack of evaluation or inconclusive reports about infectious agents, which limits the identification of predisposition for particular pathogens.
(b) The heterogeneity of reported PMM2-CDG glycan profiles and the impact of factors and modifiers like genetic polymorphisms and diet regimens hampers the establishment of direct causal relationships between altered glycosylation and infections.
(c) The multi-system involvement of most CDG and the interrelation between organs and systems poses challenges in drawing clear conclusions since patients’ immunological manifestations depend on several contexts. Uncertainty exists on whether infection recurrence is influenced by a predisposition to certain microorganisms, or by a dysfunctional immune system unable to resolve infections. Additionally, immune dysfunction in CDG may be a hallmark of the disease’s pathological mechanisms, or be secondary to other clinical manifestations (e.g., hypogammaglobulinemia subjacent to nephrotic syndrome and/or proteinuria) (164), disease implications (e.g., protein misfolding or instability) (218) or metabolic decompensation (215).

While our review explores connections between modified glycan patterns, using PMM2-CDG as a model, and the host-pathogen adhesion processes, this relationship remains a crucial area of research in the broad field of infectiology. Despite decades of research on host-pathogen interactions, the diversity and variability of glycans and adhesins in various pathogens keeps this topic active within the scientific community. Many infectious mechanisms remain elusive, emphasizing the need for additional studies, which includes the identification of unknown adhesins and their specificity. Furthermore, the relevance of alterations in specific host-pathogen interactions is still to be determined, especially when intact interactions with unaltered host-glycans persist. This points out the complexity of host-pathogen interactions and highlights the importance of continued research in this field.

All in all, this review provides a comprehensive understanding of the immunological involvement of CDG immunodeficiencies and of PMM2-CDG. It explores a line of research towards infection susceptibility, proposing altered host glycans-pathogen interactions. To address the current gaps in our understanding of immune implications in CDG, targeted research is crucial. Improved reporting of immune events and identification of pathogens responsible for infections are key steps toward establishing these connections. But other methodologies should also be taken in account:

- Collaborative research: leveraging diverse expertise and resources from different groups, can foster more comprehensive investigations into CDG immunopathology. Such collaborations spanning multiple research centers, enable standardized measurements and depth of data collected, facilitating the validation of findings across this heterogeneous patient population. For example, longitudinal studies through natural history studies such as the one currently ongoing by the Frontiers on CDG Consortium (ClinicalTrials.gov Identifier: NCT04199000).
- Interdisciplinary approaches: these are vital for a thorough understanding of the immunological alterations in CDG. By bridging disciplines like immunology, glycobiology, bioinformatics and system biology, researchers can employ multi-omics studies (e.g., genomic, proteomic and glycomics) to improve our understanding of potential genotype-phenotype associations and the roles of glycans Particularly in the context of immunological responses and susceptibility to infections in CDG, nuclear magnetic resonance presents as a promising methodology to study protein-glycan interactions (219).
- Patient involvement: advancing research in CDG’s immunological aspects is an effort across a broad range of strategic areas. By embracing the involvement of patient advocacy groups in research planning and execution, we can ensure that the studies address the most pressing needs and concerns of those affected by CDG. For instance, collecting patient-reported outcomes and quality of life data would be extremely important to have a holistic perspective on the disease’s impact, particularly in terms of immunological episodes. Namely, in PMM2-CDG, some tools have been pinpointed (220, 221).

Ultimately, understanding the immunological implications in CDG could lead to improved care and management for affected patients, enhancing their quality of life by informing more effective and personalized treatment strategies.

## Author contributions

CP: Conceptualization, Data curation, Formal analysis, Investigation, Methodology, Visualization, Writing – original draft, Writing – review & editing. RF: Data curation, Formal analysis, Investigation, Methodology, Visualization, Writing – original draft, Writing – review & editing. PM: Data curation, Formal analysis, Investigation, Methodology, Visualization, Writing – original draft, Writing – review & editing. BP: Data curation, Formal analysis, Investigation, Methodology, Visualization, Writing – original draft, Writing – review & editing. PG: Data curation, Formal analysis, Investigation, Methodology, Visualization, Writing – original draft, Writing – review & editing. HC: Writing – review & editing, Data curation, Formal analysis, Investigation, Methodology, Visualization, Writing – original draft. MB: Data curation, Formal analysis, Investigation, Methodology, Visualization, Writing – original draft, Writing – review & editing. VF: Conceptualization, Funding acquisition, Supervision, Writing – review & editing. PV: Conceptualization, Funding acquisition, Project administration, Supervision, Writing – review & editing.
