# Supplementary material for: Revisiting the immunopathology of congenital disorders of glycosylation: an updated review
Source: Front Immunol. 2024 Mar 14;15:1350101. doi: 10.3389/fimmu.2024.1350101 (PMC10972870; doi:10.3389/fimmu.2024.1350101)
Supplement: Supplementary file 2 [file Table_1.docx]

**Supplementary Table 1** - Immune manifestations of the 12 CDG with predominant immunological involvement. Clinical and immunological data represented were obtained following a literature revision of all published articles about each CDG using PubMed as the search engine. Updated from (40).^1^

| CDG (#MIM)  n | Clinical parameters | | | Biochemical parameters | | Other  (e.g., vaccination response/allergies) | References |
| --- | --- | --- | --- | --- | --- | --- | --- |
|  | **Recurrent/Severe infections** | **Pathogens** | **Autoimmune/**  **Inflammatory signs** | **WBC counts and functional parameters** | **Immunoglobulins** |  |  |
| N-linked defects | | | | | | | |
| ALG12-CDG  (#607143)  n= 12/19 | Recurrent and/or severe infections (12/19):  RTI (10/19), including pneumonia (5/19), otitis (2/19), and ear/nose infections (1/19)  Sepsis (1/19) | Most infections identified as bacterial  Mild viral RTI (1/19)  Hemolytic *E. coli* (1/19) | Aseptic meningitis (1/19)  Necrotizing enterocolitis (1/19) | ↓ B cells (1/19) | Hypogammaglobulinemia (1/19)  ↓ IgG (9/19) with abnormal IgG N-glycans (1/19)  ↓ IgM (3/19)  ↓ IgA (1/19)  ↑ IgG (1/19) | Absent response  to diphtheria, tetanus, and *H. influenzae* vaccine (1/19)  Mother's milk intolerance (1/19) | (1–8) |
| FUT8-CDG  (#618005)  n= 8/9 | Recurrent and/or severe infections (7/9):  RTI (7/9), including pneumonia (2/9), bronchopneumonia (1/9) and pulmonary infections (1/9) accompanied by respiratory failure (apnea/  hypopnea) | NA | Reactive airway disease (1/9) | Neutropenia  (1/9) | ↓ IgG with severe hypofucosylation (2/9) | NA | (9–11) |
| JAGN1-CDG  (#616022)  n=25/26 | Recurrent and/or severe infections (25/26):  Abscesses (including skin, genital and oral) (15/26)  Upper RTI (8/26), pneumonia (13/26), otitis (8/26), ear, nose, and throat infections (4/26), aphtosis, mouth ulcers, periodontitis (5/26)  Cellulitis (5/26)  Cutaneous infections (2/26)  Sepsis (3/26)  Omphalitis (4/26)  Onycholysis (1/26)  Lymphadenitis (1/26)  Dacryocystitis (1/26)  UTI (1/26)  GI tract infections (1/26) | *E. coli (2/26)*  *Aspergillus* genus (1/26)  *P. aeruginosa* (1/26)  *Candida* genus (2/26)  *K. pneumonia* (1/26)  *Enterococcus* MDR (1/26)  *M. morganii* (1/26)  *S. aureus* (1/26)  Gram negative bacillus (1/26)  *H. influenzae* (1/26) | Gingivitis (6/26) with anti-thyroglobulin  gingivitis (1/26)  Pneumonitis (2/26)  Bronchitis (2/26)  Balanitis (1/26) Periodontopathic (1/26)  Pancolitis (1/26)  Lymphadenopathy (1/26)  ↑ CRP (1/26) | Neutropenia (25/26) with ↓ RTEs (2/26)  Monocytosis (2/26)  Lymphopenia and ↓ class switched B cells and ↓ CD4 (2/26)  Leukopenia (1/26) | Hypogammaglobulinemia (2/26)  ↓ IgA (2/26)  ↓ IgG (2/26)  ↓ IgM (2/26) | BM arrest (24/26)  Dust mite allergy (1/26)  LTT test with decreased BCG, PHA, and *Candida* response (1/26)  Non protective anti-tetanus IgG and anti-diphtheria titers (1/26) | (12–15) |
| MAGT1-CDG  (#300853, #301031)  n= 35/51 | Recurrent and/or severe infections (36/51):  RTI (36/51), including sinusitis, otitis, pharyngitis, epiglottitis and pneumonia  Skin infections (17/51), namely skin warts or condylomata acuminata  Recurrent mouth sores (11/51)  Meningoencephalitis (1/51)  Sepsis (2/51) | EBV (43/51), with persistently elevated EBV-viremia (21/51)  *M. contagiosum* (11/51)  *Streptococcus* genus *(2/51)*  *P. jirovecii (1/51)*  HPV (7/51)  HVS (6/51)  VZV (5/51)  CMV (2/51)  JCV (1/51)  Metapneumovirus (1/51)  Parainfluenza Type 1 (1/51) | Severe autoimmune cytopenia (8/51)  Guillain-Barre syndrome (5/51) Immune thrombocytopenia (4/51)  Autoimmune hepatitis (3/51)  Autoimmune hemolytic anemia (3/51)  Eosinophilic esophagitis (1/51)  Chronic bronchitis (1/51)  Episcleritis (1/51)  Alopecia partialis (1/51)  Erythema multiforme (1/51)  Lymphadenopathy (6/51) and lymphadenitis (2/51), with reactive hyperplasia of the lymphoid tissue (1/51)  Ulcerative colitis (1/51)  Suppurative osteomyelitis (1/51)  Bronchiolitis (1/51)  Kawasaki disease (1/51) | Lymphopenia (2/51)  T cell lymphopenia (4/51), ↓ CD4^+^ (25/51) and ↓ CD8^+^ (2/51)  ↓ B cells (1/51)  Lymphocytosis (3/51)  ↑ T Cells (1/51)  ↑ B cells (32/51)  ↓ CD4/CD8 ratio (30/51)  ↑ % of transitional B cells (CD24hiCD38hi) (1/51)  ↓ switched memory B cells (1/51)  ↓ class-switched plasmablasts (1/51)  CD5^+^/CD19^+^ B cell predominance (1/51)  ↓ CD27^-^IgD^-^ b cell switched (1/51)  ↓ CD27^+^IgD^+^ non-switched B cell (1/51)  ↑ B cell CD27^-^ naive (1/51)  ↑ αβDNTs (20/51)  ↓ NKG2D surface expression on NK and CD8^+^ cells (28/51)  ↑ NK (1/51)  ↓ NK cells (3/51)  Transient neutropenia (14/51) and neutropenia (3/51)  EBV-encoded RNA positive T cell muscle infiltration (1/51)  ↓ expression of CD127 within CD4^+^ cells (1/51) | Hypogammaglobulinemia (3/51)  ↓ IgA (28/51)  ↓ IgG (26/51)  ↓ IgM (1/51)  ↑ IgG (1/51)  ↑ IgE (1/51)  ↑ IgM (1/51) | Absent to ineffective response to the pneumococcal polysaccharide (8/51), diphtheria (2/51) and tetanus (2/51) vaccines  Hodgkin's lymphoma (1/51)  B cell lymphoma (3/51)  Refractory non-Hodgkin lymphoma (1/51)  Selective anti-polysaccharide antibody deficiency (1/51)  Submandibular and axillary lymphadenopathy with diffuse large B cell lymphoma upon excision (1/51) | (16–25) |
| MAN2B2-CDG  (NA)  n= 1/2 | Recurrent infections (1/2) | NA | Recurrent vasculitis (1/2)  Recurrent arthritis (1/2)  Positive rheumatoid factor (1/2) | T and B lymphopenia (1/2)  ↓ naïve CD8^+^ and CD4^+^ T cells (1/2)  ↑ terminally differentiated CD8^+^CD45RA^+^CCR7^-^ cells (1/2)  Skewed repertoire of cytotoxic T cells (1/2)  ↓ T cell proliferation (1/2)  Undetectable TRECs (1/2)  ↑ circulating plasmablasts (1/2)  ↑ dysreactive B cells (1/2) | ↓ IgM (1/2)  ↓ IgA (1/2)  ↑ IgE (1/2) |  | (26,27) |
| MOGS-CDG  (#606056)  n= 29/29 | Recurrent and/or severe infections (23/29):  RTI (11/29) including pneumonia (9/29)  Sepsis (6/29)  Meningitis (2/29)  UTI (2/29)  Osteomyelitis (2/29) Tracheostomy/port infection (2/29)  Nasolacrimal abscess (1/29)  Cellulitis (1/29)  Bacterial enteritis (1/29) | *E. coli* (2/29)  *Mycoplasma* genus (2/29)  *Serratia* genus (2/29) and *S. marcescens* (1/29)  *P. Jirovecii* (2/29)  *A. baumanii* (1/29)  *E. cloacae* (1/29)  *S. pneumoniae* (1/29)  *S. aureus* (1/29)  *Pseudomonas* genus (1/29)  *Klebsiella* genus (1/29) and *K. pneumoniae* (1/29)  *Corynebacterium* genus (1/29)  *C. difficile (1/29)*  *Achromobacter* genus (1/29)  Methicillin-susceptible *S. aureus* (1/29)  Viral origin (10/29), including rhinovirus/enterovirus (3/29,) RSV (2/29), CMV (1/29) and HPV 3 and 4 (1/29) | Enteritis (1/29)  Poor wound healing (1/29)  ↑ IL-6 (1/29)  ↑ CRP (1/29) | B and T lymphocytosis with ↓ lymphocytic proliferation (2/29)  Lymphocytosis (2/29)  ↑ B cells (2/29)  Leukocytosis (2/29)  Neutropenia (2/29)  ↓ T cells (1/29)  ↑ % of neutrophils (1/29) | Hypogammaglobulinemia (22/29)  ↓ IgA (19/29)  ↓ IgM (7/29)  ↓ IgG (13/29) with ↓ half-life with ↓ FγRIIa affinity (2/29)  EBV-IgG (1/29) | Ineffective response to the measles, mumps, rubella, and varicella vaccine (2/29)  ↓ anti-meningococcal IgG and anti-pneumococcal IgG (1/29)  ↓ haemophilus influenzae B titer (1/29)  Food allergy (2/29) | (8,28–32) |
| O-linked defects | | | | | | | |
| EXTL3-CDG  (#617425)  n=13/16 | Recurrent and/or severe infections (6/16):  Upper RTI (2/16), pneumonia and other pulmonary infections (2/16)  Sepsis (2/16)  Omphalitis (1/16) Recurrent dental caries propensity (1/16)  Oral infections (1/16) | *Klebsiella* genus (1/16)  *S. aureus* (2/16)  *Candida* genus (1/16)  Upper RTI infection identified as being of viral origin (2/16)  CMV (1/16) | Exfoliating dermatitis (Omen type) (5/16)  Chronic blepharitis (1/16)  Pneumonitis (1/16) | T^-^B^+^NK^+^ (9/16) with ↓ T cell proliferation (3/16)  Idiopathic CD4 lymphopenia with no naive T cells (2/16)  ↓ T cells (1/16)  Mild lymphopenia (1/16)  ↓ or undetectable TRECs (4/16)  Eosinophilia (7/16) | Hypogammaglobulinemia (5/16)  ↓ IgG (2/16)  ↓ IgM (1/16)  ↓ IgA (1/16)  ↑ IgE (3/16)  ↑ IgG (1/16)  ↑ IgM (1/16) | Absent response to the pertussis vaccine (1/16) | (33,34) |
| Multiple glycosylation pathways defects | | | | | | | |
| ATP6AP1-CDG  (#300972)  n=31/36 | Recurrent and/or severe infections (24/36):  RTI, including pneumonia, bronchiolitis, and otitis (11/36)  Sepsis (2/36)  Plantar abscesses (5/36)  GI infections (5/36)  UTI (1/36)  Ascites (2/36)  Bacteriemia (1/36)  Peritonitis (1/36)  Cholangitis (1/36) | *E. coli* (2/36)  *S. pneumoniae* (1/36)  RSV (1/36)  SARS-CoV-2 (1/36)  Several viral infections reported in one patient (1/36) | Lymphadenopathy (1/36)  Diffuse active colitis (1/36)^a^  Gastritis (1/36)^a^  Dyspeptic syndrome associated with fever (1/36)^a^  Idiopathic hepatitis (1/36)^a^  Febrile seizure (1/36)  ↑ CRP (2/36)^a^ | Lymphopenia (1/36)  ↓ CD4^+^ and CD8^+^ T cells (1/36)  ↓ IgD1/CD27^+^ intermediate and switch memory cells indicative of defective B cell differentiation (2/36)  Leukopenia (6/36)  Neutropenia (1/36)  Pancytopenia (2/36)  Lipid-laden macrophages in liver biopsy (2/36)  Vacuolated lymphocytes (1/36) | Hypogammaglobulinemia (22/36)  ↓ IgG (12/36)  ↓ IgM (7/36)  ↓ IgA (5/36) | Ineffective response to the measles, mumps, rubella, and varicella vaccine (1/36)  Some patients responded very poorly to vaccination^b^, related to specific anti-polysaccharide antibody deficiency (1/36)  Liver fibrosis with more infiltrating lymphocytes and plasma cells in the confluent area (1/36) | (35–39) |
| G6PC3-CDG  (#612541)  n= 153/153 | Recurrent and/or severe infections (137/153):  RTI (27/153), including otitis (43/153), pneumonia (35/153), pharyngitis (16/153), aphthous stomatitis (17/153), periodontal disease (6/153), dental caries (2/153), oral infections (2/153),  bronchiolitis (1/153) and tonsillitis (2/153)  Sepsis (28/153)  UTI (15/153) including bladder infections (1/153) and pyelonephritis (1/153)  Abscesses (22/153)  Cellulitis (11/153)  GI infections (2/153)  Perianal infections (1/153)  Osteomyelitis (1/153)  Paronychial nail infections (1/153),  Conjunctival infections (1/153),  Omphalitis (4/153)  Furunculosis (1/153)  Mastoiditis (1/153)  Sinusitis (2/153) | *E. coli* (7/153)  *Aspergillus* genus (3/153)  *Candida* genus (2/153)  *Pseudomonas* genus (1/153) and *P. aeruginosa* (3/153)  *S. aureus* (4/153)  *S. viridans* (1/153)  *K.* *pneumoneae*  (1/153)  *H. pylori* (1/153)  *C. difficile* (1/153)  VZV (2/153)  RSV (1/153)  Parainfluenza (1/153)  HSV  (1/153) | IBD, including Crohn’s disease (25/153)  Colitis (5/153)  Gastroenteritis (24/153)  Oral ulcers (20/153)  Gingivitis (9/153)  Recurrent proctitis (1/153)  Panniculitis (3/153)  Gastritis (4/153) Typhlitis (1/153)  Celiac disease (1/31)  Esophagitis (1/153)  Blepharitis (1/153)  Endocarditis (1/153)  Rhinitis (1/153) Bronchitis (17/153)  Parotitis (2/153)  Recurrent fever (8/153)  Pneumonitis (3/153)  Genital aphthous ulcerations (4/153)  Eczematous rash (1/153)  Transient presence of anti-neutrophil and anti-HLA antibodies (1/153)  Erythema nodosum (1/153)  Familial Mediterranean Fever episodes (2/153)  Necrotizing enterocolitis (1/153)  Juvenile rheumatoid arthritis (2/153)  ↑ CRP (2/153) | Neutropenia (150/153) with  ↑ neutrophil ER stress (5/153), hypogranular neutrophils with rare döhle bodies (1/153) and impaired neutrophil motility and activity (1/153)  Monocytosis (6/153)  Leukopenia (17/153)  Lymphopenia (25/153) with ↓ Pan-T cell (2/153)  ↓ CD3^+^ cells (3/153)  ↓ CD4^+^ cells (8/153)  ↓ naive T cells (7/153),  ↓ B cells (8/153)  ↓ RTEs (3/153)  ↓ NK cells (5/153)  ↑ NK cells (5/153) ↑CD16^+^ and CD56^+^ (1/153)  Pancytopenia (2/153)  ↓ proliferation after mitogen stimulation (3/153)  ↑ IL-1β and IL-6 (2/153) following LPS stimulation  ↑ TNFα (on anti-TNFα therapy) (1/153) | ↑ IgG (22/153), namely during flares  ↑ IgM (2/153)  ↑ IgA (1/153) Hypogammaglobulinemia (2/131)  ↓ IgE (3/153)  ↓ IgA (1/153)  ↓ IgG (2/153) | BM maturation (55/153) with arrest at the stage of promyelocyte/ myelocyte (20/153)  BM hypercellularity (8/153)  Asthma (5/153)  Ineffective response to the tetanus (1/153) and H. influenza vaccines (1/153)  Cow milk allergy (1/153)  Medullar aplasia (1/153)  Papillary thyroid carcinoma (1/153) | (40–57) |
| PGM3-CDG  (#615816)  n=61/62 | Recurrent and/severe infections (61/62):  RTI (41/62), including pneumonia (22/62), chronic sinopulmonary disease (7/62), otitis (21/62) with tympanic membrane perforation (1/62)  Skin infections (42/62), namely abscesses (16/62), impetigo, infected skin ulcers (3/62), eczema (10/62) and oral (7/62) infections  Viral infections (14/62), including severe varicella (2/62),  Soft tissue infections (7/62), including abscesses (9/62)  Sepsis (7/62)  Perichondritis (1/62) Osteomyelitis (1/62)  Intrauterine bacterial infection (1/62)  UTI (1/62)  GI tract infections (2/62) | *Staphylococcus* genus (14/62), including *S. aureus* (6/62) and *S. epidermidis* (2/62),  *Candida* genus (19/62)  *Pseudomonas* genus (5/62)  *K. pneumoniae* (1/62)  *S. pneumoniae* (1/62)  *E. cloacae* (1/62)  *S. dysgalactiae equisimilis* (1/62)  *Salmonella* genus  (1/62)  VZV (6/62)  RSV (4/62)  EBV (3/62)  HPV (4/62)  HVS (1/62)  Influenza (1/62) | Eczema/dermatitis (40/62) often with pyodermatitis (15/62)  Rhinitis (10/62)  Bronchitis (4/62)  Esophagitis (1/62)  TSH receptor and TPO  autoantibodies (1/62)  Folliculitis (1/62)  Autoimmune hemolytic anemia (1/62)  Pericarditis (1/62)  Dorsal skin inflammation (1/62)  Conjunctivitis (1/62)  Graft versus host disease (1/62)  Bullous pemphigoid (1/62)  Blistering skin lesions (2/62)  Atopic dermatitis (2/62)  ↑ CRP (1/62) | Leukopenia (14/62)  Leukocytosis (3/62)  Neutropenia (15/62)  Neutrophilia (2/62)  Eosinophilia (28/62)  Lymphopenia (38/62), including:  ↓ T, B and NK cells (3/62)  T^-^B^-^NK^+^ (3/62)  ↓ CD3^+^ (13/62), CD4^+^ (24/62) and CD8^+^ (11/62) cells  Reverted CD4/CD8 ratio (20/62)  Th2 predominance and ↑ activated effector cells (7/62)  ↓ naïve T cells and TRECs (5/62)  Abnormal TRECs (1/62)  ↓ % of CD4^+^CD45RA^+^ T cells (1/62)  ↑ % of CD4^+^CD45RO^+^ memory T cells (1/62)  ↓ T cell proliferation (16/62)  ↓ CD45^+^ total lymphocyte proliferation (1/62)  ↓ NK cells (3/62)  ↑ NK (8/62)  ↓ B cells (19/62)  ↓ CD20^+^CD27^+^ cells (7/62)  ↑ transitional B cells (5/62) | Hypogammaglobulinemia (1/62)  ↓ IgE (1/62)  ↓ IgM (5/62)  ↓ IgA (3/62)  ↓ IgG (2/62)  ↑ IgE (46/62)  ↑ IgM (5/62)  ↑ IgG (7/62)  ↑ IgA (9/62) | Hypocellular BM (4/62)  Defective T cell development with ↑ thymic DNTs (1/62)  Asthma (7/62)  Food allergies (20/62), including cow milk (4/62)  Drug allergies (3/62)  Inadequate responses to the 23-valent pneumococcal polysaccharide vaccine (1/62)  Respiratory related allergy (7/62)  Other allergies (6/62) | (40,58–66) |
| SLC35C1-CDG  (#266265)  n= 20/20 | Recurrent and/or severe infections (17/20) although their severity and recurrence was widely variable:  RTI and pulmonary infections (11/20), namely pneumonia (3/20), bronchiolitis (1/20), otitis media (7/20), sinus infection (1/20), tonsillitis (1/20), mild to severe chronic periodontitis (8/20), recurrent dental cavities (2/20)  Severe and/or localized cellulitis (5/20)  Conjunctivitis (1/20)  UTI (1/20)  Omphalitis (1/20)  Abscesses (1/20)  Impetigo (1/20) | Coronavirus (1/20)  HSV (2/20)^c^  Also, in (1/20) infections were identified as being bacterial | Gastroenteritis (2/20)  Inflammatory skin disease (1/20)  Celiac disease (1/20) Aseptic meningitis (1/20)  Recurrent unexplained fever episodes (4/20)  ↑ CRP (1/20) | Leukocytosis (11/20)  Neutrophilia (7/20) with deficient granulation (1/20)  ↑ B and T cells (1/20)  ↓ neutrophilic mobility (6/20) | IgG defective fucosylation (1/20)  ↑ IgA and IgM (1/20) | Absent sLe^x^ and H antigen on erythrocytes (Bombay blood group) (8/20)  Egg allergy (1/20) | (8,67,68) |
| VPS13B-CDG  (#216550)  n=169/284^d^ | Recurrent and/or severe infections (63/284):  Recurrent oral infections (46/284), including aphthous ulcers and gingivitis/periodontitis  Recurrent RTI (25/284), including ear, nose and throat (13/284), pneumonia (7/284), bronchitis (1/284), meningitis (1/284), ethmoiditis (1/284), and rhinopharyngeal infections (1/284)  Skin infections (8/284), including skin warts (4/284) and cellulitis (1/284)  Stomach infection (1/284)  UTI (3/284) including pyelonephritis (1/284) | NA | Juvenile rheumatoid arthritis (1/284)  IBD (1/284)  Lichen planus (1/284) | Neutropenia (150/284)  Leukopenia (11/284) Monocytopenia (4/284)  Monocytosis (1/284)  Pancytopenia (1/284) | NA | No BM alterations were found in any of the studied patients  Positive for anti-cardiolipin IgG and anti-beta-2-glycoprotein 1 IgG (1/284) | (40,69–88) |

^1^ ↓, low or decreased; ↑, high or increased; BM, bone marrow; CMV, cytomegalovirus; CRP, C-reactive protein; DNTs, double negative T cells; EBV, Epstein-Barr virus; ER, endoplasmic reticulum; GI, gastrointestinal; HLA, human leukocyte antigen; HPV, human papilloma virus; HVS, herpes virus simplex; IBD, inflammatory bowel disease; Ig, immunoglobulin; IL, interleukin; JCV, John Cunningham virus; LPS, lipopolysaccharide; LTT, lymphocyte transformation test; MDR, multi-drug resistant; n, number of patients with immunological involvement/total of patients; NA, not available; NK, natural killer; NKG2D, natural killer group 2, member D; PHA, phytohemagglutinin; RTI, respiratory tract infections; RSV, respiratory syncytial virus; RTEs, recent thymic emigrant; sLe^X^, sialyl Lewys X; TPO, thyroid peroxidase antibodies; TNF, tumor necrosis factor; TRECs, T cell receptor excision circles; TSH, thyroid stimulating hormone; UTI, urinary tract infections; VZV, varicella zoster virus; WBC, whole blood counts

^a^ Complication found in the patient reported by [38] during immunosuppressive treatment following liver transplantation.

^b^ [39] does not disclose the exact number of patients who did not respond to vaccination

^c^ In SLC35C1-CDG patients, most often no pathogens could be identified during infection/fever episodes.

^d^ The most often intermittent nature of the neutropenia found in VPS13B-CDG patients in addition to the fact that often blood cell counts are not always reported or available but also because of the generalized lack of severe and/or recurrent infections in these patients makes it highly likely that immunological manifestations, especially neutropenia, are underdiagnosed in VPS13B-CDG.

**References**

1. Nicotera AG, Spoto G, Calì F, Romeo G, Musumeci A, Vinci M, Fiumara A, Barone R, Di Rosa G, Musumeci SA. A Novel Homozygous ALG12 Mutation in a Patient with CDG Type Ig: New Report of a Case with a Mild Phenotype. *Mol Syndromol* (2021) 12:327–332. doi: 10.1159/000516606

2. Ziburová J, Nemčovič M, Šesták S, Bellová J, Pakanová Z, Siváková B, Šalingová A, Šebová C, Ostrožlíková M, Lekka DE, et al. A novel homozygous mutation in the human ALG12 gene results in an aberrant profile of oligomannose N-glycans in patient’s serum. *Am J Med Genet A* (2021) 185:3494–3501. doi: 10.1002/AJMG.A.62474

3. Lekka D, Brucknerova J, Salingova A, Sebova C, Ostrozlikova M, Ziburova J, Nemcovic M, Sestak S, Bellova J, Pakanova Z, et al. Congenital disorders of glycosylation - an umbrella term for rapidly expanding group of rare genetic metabolic disorders - importance of physical investigation. *Bratisl Lek Listy* (2021) 122:190–195. doi: 10.4149/BLL_2021_030

4. Hiraide T, Wada Y, Matsubayashi T, Kadoya M, Masunaga Y, Ohkubo Y, Nakashima M, Okamoto N, Ogata T, Saitsu H. Novel ALG12 variants and hydronephrosis in siblings with impaired N-glycosylation. *Brain Dev* (2021) 43:945–951. doi: 10.1016/j.braindev.2021.05.013

5. de la Morena-Barrio ME, Sabater M, de la Morena-Barrio B, Ruhaak RL, Miñano A, Padilla J, Toderici M, Roldán V, Gimeno JR, Vicente V, et al. ALG12-CDG: An unusual patient without intellectual disability and facial dysmorphism, and with a novel variant. *Mol Genet Genomic Med* (2020) 8: doi: 10.1002/MGG3.1304

6. Sturiale L, Bianca S, Garozzo D, Terracciano A, Agolini E, Messina A, Palmigiano A, Esposito F, Barone C, Novelli A, et al. ALG12-CDG: novel glycophenotype insights endorse the molecular defect. *Glycoconj J* (2019) 36:461–472. doi: 10.1007/S10719-019-09890-2

7. Tahata S, Gunderson L, Lanpher B, Morava E. Complex phenotypes in ALG12-congenital disorder of glycosylation (ALG12-CDG): Case series and review of the literature. *Mol Genet Metab* (2019) 128:409–414. doi: 10.1016/J.YMGME.2019.08.007

8. Monticelli M, Ferro T, Jaeken J, dos Reis Ferreira V, Videira PA. Immunological aspects of congenital disorders of glycosylation (CDG): a review. *J Inherit Metab Dis* (2016) 39:765–780. doi: 10.1007/S10545-016-9954-9

9. Park JH, Reunert J, He M, Mealer RG, Noel M, Wada Y, Grüneberg M, Horváth J, Cummings RD, Schwartz O, et al. L-Fucose treatment of FUT8-CDG. *Mol Genet Metab Rep* (2020) 25:100680. doi: 10.1016/J.YMGMR.2020.100680

10. Ng BG, Dastsooz H, Silawi M, Habibzadeh P, Jahan SB, Fard MAF, Halliday BJ, Raymond K, Ruzhnikov MRZ, Tabatabaei Z, et al. Expanding the molecular and clinical phenotypes of FUT8-CDG. *J Inherit Metab Dis* (2020) 43:871–879. doi: 10.1002/JIMD.12221

11. Ng BG, Xu G, Chandy N, Steyermark J, Shinde DN, Radtke K, Raymond K, Lebrilla CB, AlAsmari A, Suchy SF, et al. Biallelic Mutations in FUT8 Cause a Congenital Disorder of Glycosylation with Defective Fucosylation. *Am J Hum Genet* (2018) 102:188–195. doi: 10.1016/J.AJHG.2017.12.009

12. Olcay L, Yetgin S, Erdemli E, Germeshausen M, Aktaş D, Büyükaşik Y, Okur H. Congenital dysgranulopoietic neutropenia. *Pediatr Blood Cancer* (2008) 50:115–119. doi: 10.1002/PBC.20877

13. Olcay L, Ünal Ş, Onay H, Erdemli E, Öztürk A, Billur D, Metin A, Okur H, Yıldırmak Y, Büyükaşık Y, et al. Both Granulocytic and Non-Granulocytic Blood Cells Are Affected in Patients with Severe Congenital Neutropenia and Their Non-Neutropenic Family Members: An Evaluation of Morphology, Function, and Cell Death. *Turk J Haematol* (2018) 35:229–259. doi: 10.4274/TJH.2017.0160

14. Hassanzadeh S, Sadeghi S, Jafari M, Najafi S, Molavi N, Sherkat R. Ciliary and immune dysfunctions and their genetic background in patients with non-cystic fibrosis bronchiectasis in Central Iran. *Ir J Med Sci* (2023) 192:277–283. doi: 10.1007/S11845-022-02994-Z

15. Hojabri M, Farsi Y, Jamee M, Abolhassani H, Khani HHK, Karimi A, Mesdaghi M, Chavoshzadeh Z, Sharafian S. JAGN1 mutation with distinct clinical features; two case reports and literature review. *BMC Pediatr* (2023) 23: doi: 10.1186/S12887-023-04024-Y

16. Chen S, Wang X, Sun C, Zhao CB, Lin J. MAGT1 Gene Mutation is Associated with Myositis and CD127 Expression Downregulation. *J Clin Immunol* (2023) 43:315–318. doi: 10.1007/S10875-022-01384-5/FIGURES/1

17. Watson CM, Nadat F, Ahmed S, Crinnion LA, O’Riordan S, Carter C, Savic S. Identification of a novel MAGT1 mutation supports a diagnosis of XMEN disease. *Genes Immun* (2022) 23:66. doi: 10.1038/S41435-022-00166-8

18. Bąbol-Pokora K, Wołowiec M, Popko K, Jaworowska A, Bryceson YT, Tesi B, Henter JI, Młynarski W, Badowska W, Balwierz W, et al. Molecular Genetics Diversity of Primary Hemophagocytic Lymphohistiocytosis among Polish Pediatric Patients. *Arch Immunol Ther Exp (Warsz)* (2021) 69: doi: 10.1007/S00005-021-00635-4

19. Jalil M, Rowane M, Rajan J, Hostoffer R. Successful Anti-SARS-CoV-2 Spike Protein Antibody Response to Vaccination in MAGT1 Deficiency. *Allergy Rhinol (Providence)* (2021) 12: doi: 10.1177/21526567211056239

20. Peng X, Lu Y, Wang H, Wu B, Gan M, Xu S, Zhuang D, Wang J, Sun J, Wang X, et al. Further Delineation of the Spectrum of XMEN Disease in Six Chinese Pediatric Patients. *Front Genet* (2022) 13: doi: 10.3389/FGENE.2022.768000

21. Au EYL, Tung EKK, Ip RWK, Li PH. Novel MAGT1 Mutation Found in the First Chinese XMEN in Hong Kong. *Case Reports Immunol* (2022) 2022: doi: 10.1155/2022/2390167

22. Haskologlu S, Baskin K, Aytekin C, Islamoglu C, Ceylaner S, Dogu F, Tacyildiz N, Unal E, Ikinciogullari A. Scales of Magt1 Gene: Novel Mutations, Different Presentations. *Iran J Allergy Asthma Immunol* (2022) 21:92–97. doi: 10.18502/IJAAI.V21I1.8622

23. Guha S, Khetrapal P. Recurrent oral ulcers due to XMEN syndrome. *Sri Lanka Journal of Child Health* (2022) 51:139–141. doi: 10.4038/SLJCH.V51I1.10022

24. Huang X, Liu D, Gao Z, Liu C. Case Report: EBV-Positive Extra-Nodal Marginal Zone Lymphoma Associated With XMEN Disease Caused by a Novel Hemizygous Mutation in MAGT1. *Front Oncol* (2021) 11: doi: 10.3389/FONC.2021.653266

25. Ravell JC, Chauvin SD, He T, Lenardo M. An update on XMEN disease. *J Clin Immunol* (2020) 40:671. doi: 10.1007/S10875-020-00790-X

26. Verheijen J, Wong SY, Rowe JH, Raymond K, Stoddard J, Delmonte OM, Bosticardo M, Dobbs K, Niemela J, Calzoni E, et al. Defining a new immune deficiency syndrome: MAN2B2-CDG. *J Allergy Clin Immunol* (2020) 145:1008. doi: 10.1016/J.JACI.2019.11.016

27. Tian Q, Shu L, Shu C, Xi H, Ma N, Mao X, Wang H. Compound heterozygous variants in MAN2B2 identified in a Chinese child with congenital disorders of glycosylation. *Eur J Hum Genet* (2022) doi: 10.1038/S41431-022-01125-7

28. Anzai R, Tsuji M, Yamashita S, Wada Y, Okamoto N, Saitsu H, Matsumoto N, Goto T. Congenital disorders of glycosylation type IIb with MOGS mutations cause early infantile epileptic encephalopathy, dysmorphic features, and hepatic dysfunction. *Brain Dev* (2021) 43:402–410. doi: 10.1016/J.BRAINDEV.2020.10.013

29. Beimdiek J, Hennig R, Burock R, Puk O, Biskup S, Rapp E, Lesinski-Schiedat A, Buettner FFR, Das AM. Serum N-glycomics of a novel CDG-IIb patient reveals aberrant IgG glycosylation. *Glycobiology* (2022) 32:380–390. doi: 10.1093/GLYCOB/CWAC003

30. Abuduxikuer K, Wang L, Zou L, Cao CY, Yu L, Guo HM, Liang XM, Wang JS, Chen L. Updated clinical and glycomic features of mannosyl-oligosaccharide glucosidase deficiency: Two case reports. *World J Clin Cases* (2022) 10:7397–7408. doi: 10.12998/WJCC.V10.I21.7397

31. Shimada S, Ng BG, White AL, Nickander KK, Turgeon C, Liedtke KL, Lam CT, Font-Montgomery E, Lourenco CM, He M, et al. Clinical, biochemical and genetic characteristics of MOGS-CDG: a rare congenital disorder of glycosylation. *J Med Genet* (2022) 59:1104–1115. doi: 10.1136/JMEDGENET-2021-108177

32. Post MA, de Wit I, Zijlstra FSM, Engelke UFH, van Rooij A, Christodoulou J, Tan TY, Le Fevre A, Jin D, Yaplito-Lee J, et al. MOGS-CDG: Quantitative analysis of the diagnostic Glc3 Man tetrasaccharide and clinical spectrum of six new cases. *J Inherit Metab Dis* (2023) 46:313–325. doi: 10.1002/JIMD.12588

33. Bajaj S, Satoskar P, Nair A, Sheth F, Sheth J, Sheth H. An ultra-rare case of immunoskeletal dysplasia with neurodevelopmental abnormalities in an Indian patient with homozygous c.953C > T variant in EXTL3 gene: a case report. *BMC Pediatr* (2022) 22: doi: 10.1186/S12887-022-03143-2

34. Akalın A, Taskiran EZ, Şimşek-Kiper PÖ, Utine E, Alanay Y, Özçelik U, Boduroğlu K. Spondyloepimetaphyseal dysplasia EXTL3-deficient type: Long-term follow-up and review of the literature. *Am J Med Genet A* (2021) 185:3104–3110. doi: 10.1002/AJMG.A.62378

35. Barua S, Berger S, Pereira EM, Jobanputra V. Expanding the phenotype of ATP6AP1 deficiency. *Cold Spring Harb Mol Case Stud* (2022) 8: doi: 10.1101/MCS.A006195

36. Alharbi H, Daniel EJP, Thies J, Chang I, Goldner DL, Ng BG, Witters P, Aqul A, Velez-Bartolomei F, Enns GM, et al. Fractionated plasma N-glycan profiling of novel cohort of ATP6AP1-CDG subjects identifies phenotypic association. *J Inherit Metab Dis* (2023) 46:300–312. doi: 10.1002/JIMD.12589

37. Dang Do AN, Chang IJ, Jiang X, Wolfe LA, Ng BG, Lam C, Schnur RE, Allis K, Hansikova H, Ondruskova N, et al. Elevated oxysterol and N-palmitoyl-O-phosphocholineserine levels in congenital disorders of glycosylation. *J Inherit Metab Dis* (2023) 46:326–334. doi: 10.1002/JIMD.12595

38. Semenova N, Shatokhina O, Shchagina O, Kamenec E, Marakhonov A, Degtyareva A, Taran N, Strokova T. Clinical Presentation of a Patient with a Congenital Disorder of Glycosylation, Type IIs (ATP6AP1), and Liver Transplantation. *International Journal of Molecular Sciences 2023, Vol 24, Page 7449* (2023) 24:7449. doi: 10.3390/IJMS24087449

39. Jansen EJR, Timal S, Ryan M, Ashikov A, Van Scherpenzeel M, Graham LA, Mandel H, Hoischen A, Iancu TC, Raymond K, et al. ATP6AP1 deficiency causes an immunodeficiency with hepatopathy, cognitive impairment and abnormal protein glycosylation. *Nat Commun* (2016) 7: doi: 10.1038/NCOMMS11600

40. Pascoal C, Francisco R, Ferro T, dos Reis Ferreira V, Jaeken J, Videira PA. CDG and immune response: From bedside to bench and back. *J Inherit Metab Dis* (2020) 43:90–124. doi: 10.1002/JIMD.12126

41. Yildirmak ZY, Ozcelik G, Ozagari AA, Genc DB, Onay H. Amyloidosis in a Patient With Congenital Neutropenia Because of G6PC3 Deficiency. *J Pediatr Hematol Oncol* (2022) 44:E431–E433. doi: 10.1097/MPH.0000000000002237

42. Peruffo M V., Nainsztein G, Quiña VS, Samaruga C, Cuello MF, Romano S, Caferri H. [Congenital neutropenia type IV: case report]. *Arch Argent Pediatr* (2022) 120:E213–E217. doi: 10.5546/AAP.2022.E213

43. Dai R, Lv G, Li W, Tang W, Chen J, Liu Q, Yang L, Zhang M, Tian Z, Zhou L, et al. Altered Functions of Neutrophils in Two Chinese Patients With Severe Congenital Neutropenia Type 4 Caused by G6PC3 Mutations. *Front Immunol* (2021) 12: doi: 10.3389/FIMMU.2021.699743

44. Jeong D, Kim SM, Min BJ, Kim JH, Ju YS, Ahn YO, Yun J, Lee YE, Kwon SR, Park JH, et al. Heterogeneous genetic landscape of congenital neutropenia in Korean patients revealed by whole exome sequencing: genetic, phenotypic and histologic correlations. *Sci Rep* (2022) 12: doi: 10.1038/S41598-022-11492-2

45. Moradian N, Zoghi S, Rayzan E, Seyedpour S, Jimenez Heredia R, Boztug K, Rezaei N. Severe congenital neutropenia due to G6PC3 deficiency: early and delayed phenotype of a patient. *Allergy Asthma Clin Immunol* (2023) 19: doi: 10.1186/S13223-023-00804-4

46. Nikolouzakis TK, Spyridakis K, Tzardi M, Tsaknakis G, Ximeri M, Klimiankou M, Chrysos E, Skokowa J, Papadaki HA. Chronic neutropenic colitis with complete colonic obstruction in a patient with severe congenital neutropenia associated with G6PC3 mutations. *Ann Hematol* (2022) 101:1583–1585. doi: 10.1007/S00277-022-04772-4

47. López-Rodríguez L, Svyryd Y, Benítez-Alonso EO, Rivero-García P, Luna-Muñoz L, Mutchinick OM. Severe Congenital Neutropenia Type 4: A Rare Disease Harboring a G6pc3 Gene Pathogenic Variant Particular to the Mexican Population. *Rev Invest Clin* (2022) 74:328–339. doi: 10.24875/RIC.22000234

48. Maroufi SF, Shaka Z, Mojtabavi H, Sadeghalvad M, Rayzan E, Sedighi I, Shahkarami S, Najafi M, Rohlfs M, Klein C, et al. Novel G6PC3 Mutations in Patients with Congenital Neutropenia: Case Reports and Review of the Literature. *Endocr Metab Immune Disord Drug Targets* (2021) 21:1660–1668. doi: 10.2174/1871530321666210616110631

49. Hiwarkar P, Bargir U, Pandrowala A, Bodhanwala M, Thakker N, Taur P, Madkaikar M, Desai M. SLGT2 Inhibitor Rescues Myelopoiesis in G6PC3 Deficiency. *J Clin Immunol* (2022) 42:1653–1659. doi: 10.1007/S10875-022-01323-4

50. Boulanger C, Stephenne X, Diederich J, Mounkoro P, Chevalier N, Ferster A, Van Schaftingen E, Veiga-da-Cunha M. Successful use of empagliflozin to treat neutropenia in two G6PC3-deficient children: Impact of a mutation in SGLT5. *J Inherit Metab Dis* (2022) 45:759–768. doi: 10.1002/JIMD.12509

51. Veiga-da-Cunha M, Chevalier N, Stephenne X, Defour JP, Paczia N, Ferster A, Achouri Y, Dewulf JP, Linster CL, Bommer GT, et al. Failure to eliminate a phosphorylated glucose analog leads to neutropenia in patients with G6PT and G6PC3 deficiency. *Proc Natl Acad Sci U S A* (2019) 116:1241–1250. doi: 10.1073/PNAS.1816143116

52. Giacaman A, Salinas Sanz JA, Navarro Noguera S, Díaz de Heredia Rubio C, Martín-Santiago A. Prominent venous circulation and thick lips in an 8-year-old boy with congenital neutropenia. *Pediatr Dermatol* (2019) 36:e69–e70. doi: 10.1111/PDE.13760

53. Goenka A, Doherty JA, Al-Farsi T, Jagger C, Banka S, Cheesman E, Fagbemi A, Hughes SM, Wynn RF, Hussell T, et al. Neutrophil dysfunction triggers inflammatory bowel disease in G6PC3 deficiency. *J Leukoc Biol* (2021) 109:1147–1154. doi: 10.1002/JLB.5AB1219-699RR

54. A Novel Mutation in G6PC3 Gene Associated Non-syndromic Severe Congenital Neutropenia.

55. McKinney C, Ellison M, Briones NJ, Baroffio A, Murphy J, Tran AD, Reisz JA, D’Alessandro A, Ambruso DR. Metabolic abnormalities in G6PC3-deficient human neutrophils result in severe functional defects. *Blood Adv* (2020) 4:5888–5901. doi: 10.1182/BLOODADVANCES.2020002225

56. Dasouki M, Alaiya A, ElAmin T, Shinwari Z, Monies D, Abouelhoda M, Jabaan A, Almourfi F, Rahbeeni Z, Alsohaibani F, et al. Comprehensive multi-omics analysis of G6PC3 deficiency-related congenital neutropenia with inflammatory bowel disease. *iScience* (2021) 24: doi: 10.1016/J.ISCI.2021.102214

57. Velez-Tirado N, Yamazaki-Nakashimada MA, Lopez Valentín E, Partida-Gaytan A, Scheffler-Mendoza SC, Chaia Semerena GM, Alvarez-Cardona A, Suárez Gutiérrez MA, Medina Torres EA, Baeza Capetillo P, et al. Severe congenital neutropenia due to G6PC3 deficiency: Case series of five patients and literature review. *Scand J Immunol* (2022) 95:e13136. doi: 10.1111/SJI.13136

58. Lundin KE, Wang Q, Hamasy A, Marits P, Uzunel M, Wirta V, Wikström AC, Fasth A, Ekwall O, Smith CIE. Eleven percent intact PGM3 in a severely immunodeficient patient with a novel splice-site mutation, a case report. *BMC Pediatr* (2018) 18: doi: 10.1186/S12887-018-1258-9

59. Ben-Ali M, Ben-Khemis L, Mekki N, Yaakoubi R, Ouni R, Benabdessalem C, Ben-Mustapha I, Barbouche MR. Defective glycosylation leads to defective gp130-dependent STAT3 signaling in PGM3-deficient patients. *J Allergy Clin Immunol* (2019) 143:1638-1640.e2. doi: 10.1016/J.JACI.2018.12.987

60. Ittiwut C, Manuyakorn W, Tongkobpetch S, Benjaponpitak S, Fisher MR, Milner JD, Lyons JJ, Suphapeetiporn K, Shotelersuk V. Compound Heterozygous PGM3 Mutations in a Thai Patient with a Specific Antibody Deficiency Requiring Monthly IVIG Infusions. *J Clin Immunol* (2020) 40:227–231. doi: 10.1007/S10875-019-00693-6

61. Fusaro M, Vincent A, Castelle M, Rosain J, Fournier B, Veiga-da-Cunha M, Kentache T, Serre J, Fallet-Bianco C, Delezoide AL, et al. Two Novel Homozygous Mutations in Phosphoglucomutase 3 Leading to Severe Combined Immunodeficiency, Skeletal Dysplasia, and Malformations. *J Clin Immunol* (2021) 41:958–966. doi: 10.1007/S10875-021-00985-W

62. García-García A, Buendia Arellano M, Deyà-Martínez À, Lozano Blasco J, Serrano M, Van Den Rym A, García-Solis B, Esteve-Solé A, Yiyi L, Vlagea A, et al. Novel PGM3 compound heterozygous variants with IgE-related dermatitis, lymphopenia, without syndromic features. *Pediatr Allergy Immunol* (2021) 32:566–575. doi: 10.1111/PAI.13398

63. Garib V, Ben-Ali M, Kundi M, Curin M, Yaakoubi R, Ben-Mustapha I, Mekki N, Froeschl R, Perkmann T, Valenta R, et al. Profound differences in IgE and IgG recognition of micro-arrayed allergens in hyper-IgE syndromes. *Allergy* (2022) 77:1761–1771. doi: 10.1111/ALL.15143

64. Winslow A, Jalazo ER, Evans A, Winstead M, Moran T. A De Novo Cause of PGM3 Deficiency Treated with Hematopoietic Stem Cell Transplantation. *J Clin Immunol* (2022) 42:691–694. doi: 10.1007/S10875-021-01196-Z

65. Fallahi M, Jamee M, Enayat J, Abdollahimajd F, Mesdaghi M, Khoddami M, Segarra-Roca A, Frohne A, Dmytrus J, Keramatipour M, et al. Novel PGM3 mutation in two siblings with combined immunodeficiency and childhood bullous pemphigoid: a case report and review of the literature. *Allergy Asthma Clin Immunol* (2022) 18:111. doi: 10.1186/S13223-022-00749-0

66. Jacob M, Masood A, Abdel Rahman AM. Multi-Omics Profiling in PGM3 and STAT3 Deficiencies: A Tale of Two Patients. *Int J Mol Sci* (2023) 24: doi: 10.3390/IJMS24032406

67. Tahata S, Raymond K, Quade M, Barnes S, Boyer S, League S, Kumanovics A, Abraham R, Jacob E, Menon P, et al. Defining the mild variant of leukocyte adhesion deficiency type II (SLC35C1-congenital disorder of glycosylation) and response to l-fucose therapy: Insights from two new families and review of the literature. *Am J Med Genet A* (2022) 188:2005–2018. doi: 10.1002/AJMG.A.62737

68. Dyment DA, O’Donnell-Luria A, Agrawal PB, Coban Akdemir Z, Aleck KA, Antaki D, Al Sharhan H, Au PYB, Aydin H, Beggs AH, et al. Alternative genomic diagnoses for individuals with a clinical diagnosis of Dubowitz syndrome. *Am J Med Genet A* (2021) 185:119–133. doi: 10.1002/AJMG.A.61926

69. Zhao S, Luo Z, Xiao Z, Li L, Zhao R, Yang Y, Zhong Y. Case report: two novel VPS13B mutations in a Chinese family with Cohen syndrome and hyperlinear palms. *BMC Med Genet* (2019) 20: doi: 10.1186/S12881-019-0920-X

70. Duplomb L, Rivière J, Jego G, Da Costa R, Hammann A, Racine J, Schmitt A, Droin N, Capron C, Gougerot-Pocidalo MA, et al. Serpin B1 defect and increased apoptosis of neutrophils in Cohen syndrome neutropenia. *J Mol Med (Berl)* (2019) 97:633–645. doi: 10.1007/S00109-019-01754-4

71. Boschann F, Fischer-Zirnsak B, Wienker TF, Holtgrewe M, Seelow D, Eichhorn B, Döhnert S, Fahsold R, Horn D, Graul-Neumann LM. An intronic splice site alteration in combination with a large deletion affecting VPS13B (COH1) causes Cohen syndrome. *Eur J Med Genet* (2020) 63: doi: 10.1016/J.EJMG.2020.103973

72. Momtazmanesh S, Rayzan E, Shahkarami S, Rohlfs M, Klein C, Rezaei N. A novel VPS13B mutation in Cohen syndrome: a case report and review of literature. *BMC Med Genet* (2020) 21: doi: 10.1186/S12881-020-01075-1

73. Rakusiewicz K, Kanigowska K, Hautz W, Wicher D, Młynek M, Wyszyńska M, Rogowska A, Jȩdrzejczak-Młodziejewska J, Danowska M, Czeszyk A. Coexistence of bilateral macular edema and pale optic disc in the patient with Cohen syndrome. *Open Medicine (Poland)* (2021) 16:156–160. doi: 10.1515/MED-2021-0208/MACHINEREADABLECITATION/RIS

74. Hennies HC, Rauch A, Seifert W, Schumi C, Moser E, Al-Taji E, Tariverdian G, Chrzanowska KH, Krajewska-Walasek M, Rajab A, et al. Allelic heterogeneity in the COH1 gene explains clinical variability in Cohen syndrome. *Am J Hum Genet* (2004) 75:138–145. doi: 10.1086/422219

75. Li L, Bu X, Ji Y, Tan P, Liu S. A Novel Homozygous VPS13B Splice-Site Mutation Causing the Skipping of Exon 38 in a Chinese Family With Cohen Syndrome. *Front Pediatr* (2021) 9: doi: 10.3389/FPED.2021.651621

76. Douzgou S, Petersen MB. Clinical variability of genetic isolates of Cohen syndrome. *Clin Genet* (2011) 79:501–506. doi: 10.1111/J.1399-0004.2011.01669.X

77. Razavi A, Jafarpour H, Khosravi M reza, Abbasi G, Dabbaghzadeh A. A VPS13B mutation in Cohen syndrome presented with petechiae: An unusual presentation. *Clin Case Rep* (2021) 9: doi: 10.1002/CCR3.4492

78. Karimzadeh MR, Omidi F, Sahebalzamani A, Saeidi K. A Novel VPS13B Mutation Identified by Whole-Exome Sequencing in Iranian Patients with Cohen Syndrome. *J Mol Neurosci* (2021) 71:2566–2574. doi: 10.1007/S12031-021-01852-4

79. Hussain A, Acharya A, Bharadwaj T, Genomics UOWCFM, Leal SM, Khaliq A, Mir A, Schrauwen I. A Novel Variant in VPS13B Underlying Cohen Syndrome. *Biomed Res Int* (2023) 2023: doi: 10.1155/2023/9993801

80. Daich Varela M, Motta FL, Webster AR, Arno G. A rare canonical splice-site variant in VPS13B causes attenuated Cohen syndrome. *Ophthalmic Genet* (2022) 43:110–115. doi: 10.1080/13816810.2021.1970194

81. Hu X, Huang T, Liu Y, Zhang L, Zhu L, Peng X, Zhang S. Identification of a Novel VPS13B Mutation in a Chinese Patient with Cohen Syndrome by Whole-Exome Sequencing. *Pharmgenomics Pers Med* (2021) 14:1583–1589. doi: 10.2147/PGPM.S327252

82. Ishikawa E, Shibuya M, Kimura Y, Kamekura N, Fujisawa T. A Cohen syndrome patient whose muscle-relaxant effect may have been prolonged during general anesthesia: a case report. *J Dent Anesth Pain Med* (2022) 22:155. doi: 10.17245/JDAPM.2022.22.2.155

83. Sevik MO, Aykut A, Şahin Ö. Resolution of cystoid macular edema with topical carbonic anhydrase inhibitor in a patient with retinal dystrophy associated with Cohen syndrome. *Ophthalmic Genet* (2021) 42:619–623. doi: 10.1080/13816810.2021.1925928

84. Dehghan R, Behnam M, Moafi A, Salehi M. A Novel Mutation in the VPS13B Gene in a Cohen Syndrome Patient with Positive Antiphospholipid Antibodies. *Case Reports Immunol* (2021) 2021: doi: 10.1155/2021/3143609

85. AbdelAleem A, Haddad N, Al-Ettribi G, Crunk A, Elsotouhy A. Cohen syndrome and early-onset epileptic encephalopathy in male triplets: two disease-causing mutations in VPS13B and NAPB. *Neurogenetics* (2023) 24:103–112. doi: 10.1007/S10048-023-00710-2

86. Gong J, Zhang L, Long Y, Xiao B, Long H. Cohen syndrome in two patients from China. *Mol Genet Genomic Med* (2022) 10: doi: 10.1002/MGG3.2053

87. Moosa S, Chentli F, Altmüller J, Bögershausen N, Nürnberg P, Yigit G, Li Y, Wollnik B. Genomic basis of syndromic short stature in an Algerian patient cohort. *Am J Med Genet A* (2022) 188:606–612. doi: 10.1002/AJMG.A.62532

88. Güneş N, Alkaya DU, Demirbilek V, Yalçınkaya C, Tüysüz B. Early Diagnostic Signs and the Natural History of Typical Findings in Cohen Syndrome. *J Pediatr* (2023) 252:93–100. doi: 10.1016/J.JPEDS.2022.08.052
